# Supplementary material for: Ectomycorrhizal access to organic nitrogen mediates CO2 fertilization response in a dominant temperate tree
Source: Nat Commun. 2021 Sep 13;12:5403. doi: 10.1038/s41467-021-25652-x (PMC8438073; doi:10.1038/s41467-021-25652-x)
Supplement: Supplementary file 1 — Supplementary Information [file 41467_2021_25652_MOESM1_ESM.pdf]

Supplementary Information for: ***Ectomycorrhizal access to organic nitrogen mediates CO_2 fertilization response in a dominant temperate tree***

Peter T. Pellitier, Inés Ibáñez, Donald R. Zak, William A. Argiroff, Kirk Acharya

This PDF file includes:

Supplementary Text: Methods
Supplementary Figures 1-14
Supplementary Tables 1-8
Supplementary References

Supplementary Methods:
Five soil cores, 5-cm diameter and 10-cm deep, were collected both May and August 2018, were taken radially around the dripline of each focal *Q. rubra* individual. Cores were immediately transferred to the laboratory on ice, sieved at 2 mm, and homogenized for each tree.
Net N mineralization rates were calculated by averaging technical replicates, and summing the concentrations of NO_3^- and NH_4^+ prior to and following incubation; net N mineralization was the difference between these values[1]. All analyses presented here utilize August 2018 rates of net N mineralization due to several May incubations being disturbed. Total free primary amines (TFPA) in soil (primarily amino acids and amino sugars) was measured using fresh sieved soil extracted with 2M KCl. TFPA is expressed as μmol leucine equivalents, because leucine was used as the analytical standard estimates of TFPA availability may be considered relative indices of labile organic N availability in soil solution, which is distinct from N-SOM. Extracts used for TFPA quantification were identical to those used to measure extractable NO_3^- and NH_4^+ prior to aerobic incubation. Contents of dried sieved bulk soil (% of dry mass) were determined using combustion analysis on a LECO TruMac CN analyzer (LECO Corporation, St. Joseph, MI, USA) for soil collected in May 2018. Soil pH was determined for 2:1 deionized water-soil slurries with an Accumet 15 pH meter for soil collected in August 2018 (Fisher Scientific, Waltham, MA, USA). See [2] for all values.

Fungal cores were obtained immediately adjacent to the soil cores described above. ECM cores were pooled for each individual focal tree and within 12 days of sampling, definitive ECM root-tips with high turgor were manually excised using a dissecting microscope after visually eliminating non-*Quercus* roots. Sampling was standardized by visually assessing the tips of ~ 90% (wet weight) of all *Quercus* roots in each of the root cores.

Molecular characterization of ECM communities is described in [2]. CTAB from each ECM root-tip sample was removed prior to lyophilization at -50°C. Lyophilized root-tips were homogenized and the totality of each root-tip sample was split into 20-25mg components and each component was placed in a lysis-tube with three sterilized 2.38mm metal beads. Each lysis tube also contained 800μl of Buffer AP1 and 4μl of RNase A from a Qiagen DNeasy Plant Mini Kit. Tubes were vortexed and placed in a 65°C waterbath for 20 minutes. DNA was then extracted using the Qiagen DNeasy Plant Mini Kit following manufacturers recommended protocol. DNA extraction replicates were combined and DNA yield was assessed using gel electrophoresis. Assessment of DNA quality was conducted using a Nanodrop Spectrophotometer (Thermo Fisher). The Quant-iT PicoGreen dsDNA Assay Kit (LifeTechnologies) and a BioTek SynergyHT Multi-Detection Microplate Reader (BioTek Instruments) were used to quantify DNA concentrations prior to PCR.

The ITS2 region was amplified using Illumina dual-indexed primers 5.8S Fun and ITS4 Fun [3]. The forward and reverse primer each contained the appropriate Illumina Nextera adaptor, linker sequence and error correcting Golay barcode for use with the Illumina MiSeq platform. All PCRs were performed in triplicate following Taylor et al. (2016), using Phusion High Fidelity DNA Polymerase and master mix (New England BioLabs). Each PCR contained 6 μl High Fidelity Phusion 5 × buffer, 0.75 μl each primer (10 μm initial concentration), 0.42 μl dNTPs (20 $mmol^{-1}$ initial concentration of each dNTP), 1.5 μl of template DNA (mean concentration 3.76 ng/μl, SD=2.82) and 0.23 μl of Taq (2 U/μl) brought to a final volume of 20 μl with molecular-grade water. PCR conditions consisted of an initial denaturation step at 94°C for 3 min, followed by 27 cycles of the following: 30 s at 94°C, 45 s at 57°C and 90 s at 72°C followed by a final extension step of 72°C for 10 min. PhiX oligonucleotides were spiked for

base diversity.

Taxonomic identification of fungi encountered in this study are described in full elsewhere [2]. The DADA2 pipeline was implemented in QIIME 2 in order to denoise sequences, detect and remove chimeras and remove PhiX contaminants and infer representative sequences named absolute sequence variants (ASV) [4]. A maximum of 2 expected errors (MaxEE = 2) was allowed. ASV were inferred using a total 6,869,462 of filtered forward sequences (mean= 5.64 x 10^5, *SD*= 1.39 x 10^5 sequences per sample). ASV were assigned taxonomy using the dynamic (97-99% sequence similarity) UNITE database (v.8)[5] and the scikit-learn naive Bayes machine-learning algorithm [6]. This dynamic classification system captures known variation among fungal clades in delimited species sequence similarity [7]. ASV were collapsed at the finest possible taxonomic level using the *taxa collapse* command in QIIME 2. Taxa that could not be assigned to Fungi, and appeared less than twice across all samples were removed.

After filtering and processing, sequence-based rarefaction curves were highly asymptotic implying that sequencing depth was adequate to capture the diversity of fungi encountered in our samples[2]. The ectomycorrhizal status of fungal genera was assigned using literature searches[8]; fungal genera with mixed, unidentified, or non-ECM status were removed from subsequent analyses (~20% of overall sequences). Individual root-systems hosted a mean of 27 ECM OTU (SE = 0.95), and an average of 80% sequences per sample consisted of ECM taxa (SE = 0.020%). This is almost certainly a slight under-estimation of per-sample ECM sequence abundance, because fungal taxa with questionable or uncertain biotrophic associations, although detected from ECM root-tips samples, were not scored as ECM [9].

We used the DEEMY (characterization and DEtermination of EctoMYcorrhizae) database (http://www.deemy.de/) to gather trait information on the exploration type (hyphal foraging distance) and rhizomorph formation of ECM fungal species present in our dataset. When fungal species in our study were not represented in DEEMY, congeners were surveyed and, if 90% of the entries agreed, consensus trait values were assigned to that taxon. This classification system is supported by the fact that foraging-related functional traits for fungal hyphae are typically conserved at the genus level [10]. This also allowed incorporation of ECM taxa that could only be identified to genus [11]. Long-distance foraging types were rare in our study system, composing less than 7% of ECM-derived sequences in each sample (SE = 1.38) and were removed from subsequent analyses. We were able to assign morphological hyphal trait data for 28 ECM genera comprising more than 93% all identified ECM sequences. The proportion of ECM sequences assigned morphological attributes using DEEMY did not vary across the soil gradient (P = 0.50).

Threshold Indicator Analysis was used to detect ECM community level threshold responses – community change points- along the continuous soil N mineralization gradient, using the TITAN2 package in R [12]. Only validated ECM genera were used in this analysis, and taxa that occurred in less than five samples, less than five times per sample were removed following [12]. Community-level changes are strongest where either sum (z−) or sum (z+) reaches a maximum. Evidence for a community-level threshold is obtained when (a) many species exhibit similar change points, (b) a large maximum z-score occurs relative to sums elsewhere on the gradient and (c) z-score maxima across bootstrap replicates occupy a relatively narrow range of environmental values. We conducted this analysis after Hellinger transformation using 2000 bootstrap and permutation replicates. We used relative cutoff and threshold scores of 0.85, Indvals were calculated using the relative abundance obtained by the ratio of summed abundance in each partition to the total, to address skew.

Prior to metagenomic sequencing library preparation, DNA extracts were quantified (Agilent 4200 TapeStation; Santa Clara, CA). 40ng of input DNA was used for library construction, however six of the 60 samples had lower total DNA yield. For these samples, the totality of all DNA was used. Libraries were then custom sheared using a Covaris S2 Focused-Ultrasonicator Woburn, MA), to a target of 200 bp (duty =10%, intensity = 5, cycles/burst = 200, time =120 seconds); previous trials confirmed the efficacy of these settings. Libraries were prepared using the NEB Next Ultra 2 DNA Library Prep kit (New England Biolabs) with seven cycles of PCR. 59 out of the 60 samples successfully yielded libraries suitable for sequencing. Sequencing was conducted using an S4 flow cell of the Illumina NovaSeq 6000 instrument.

In total, 23,203,326,006 sequences were generated. Reads were then dereplicated, adapters trimmed, sequence Q >20 retained, and reads shorter than 40 bp were removed using BBDuk (jgi.doe.gov). 23,177,098,622 paired-end reads passed initial quality filtering. We then used an additional filtering step to remove non-fungal sequences using Kraken2 paired-end mode with default parameters [13]. Sequences were mapped against the standard Kraken2 database containing bacterial, archaeal and UniVec reads (containing sequencing adapters, linkers, and primer sequences), and further supplemented with sequences obtained from published *Quercus rubra* [14] and *Qurcus lobata* genomes [15] in order to remove plant sequences (contaminants). All mapped reads were removed. On average, 21.7% of sequences per sample were removed during this Kraken2 filtering step, and the mean number of sequences remaining in each sample after Kraken mapping was 307,041,274

Filtered reads were mapped to functional reference gene databases CAZy (accessed March 2019) [16] and Peroxibase (accessed February 2019)[17]. Translated reads were mapped to CAZy using 'sensitive' mode in DIAMOND v. 0.9.29, with an -e value: $1e^{-4}$, following best practices for unmerged reads [18]. BWA-MEM was used to map sequences to representatives downloaded from Peroxibase, using default settings [19]. The number of mapped reads was averaged for unmerged forward and reverse reads for each reference gene to avoid double counting, the geometric mean of all mapped reads were then averaged across all reference sequences for a given gene family. Note that gene counts were not normalized to gene size (for example, average length of each gene in CAZy) because this was unnecessary: comparisons of CAZy relative abundances were primarily to environmental parameters and not to each other.

We tabulated the number of near-single copy genes, as a proxy for the number of Dikaryotic fungal genomes present in each sample, using the OrthoDB v.9 orthologous ancestral gene database, which comprised 1312 near-single copy gene variants [20] Filtered forward and reverse reads for each sample were mapped to the Dikaryotic OrthoDB database of 1312 orthologs using 'sensitive' DIAMOND as above. Mapped reads to each ortholog were averaged to prevent double-counting. Dikaryotic near single-copy genes were chosen because the majority (>95% sequences) are Dikaryotic. Instead of relying on a single arbitrarily chosen house-keeping gene that may not be at true single-copy in complex environmental samples, we calculated the geometric mean number of 'single-copy' genes present across all orthologs and the standard error of orthologous gene counts for each sample.

The following analyses were run in JAGS 3.4 [21] using the rjags package in R (R Development Core Team 2013). Three chains were run until convergence of the parameters, ~50,000 iterations, and run again for another 50,000 to estimate posterior parameter means, variances and covariances, after thinning every 100th iteration.

BAI analysis: rjags analysis code

```
model{

#missing temp
for(i in 1:38){MayMinTemp[i]~dnorm(0,1)}

  for(i in 2:55){  #individuals
     J[i]<-step(miner[i]-cp)

    for(y in 72:109){  #years 1980-2017

    bai[y,i]~dlnorm(D[y,i],tau1[y,i])
    bai.h[y,i]~dlnorm(D[y,i],tau1[y,i])#predicted
    D[y,i]<- (alpha[1]+alpha[2]*J[i])+(alpha[3]+alpha[4]*J[i])*(miner[i])+alpha[5]*log(dbh[y,i])+
alpha[6]**baiS[y-1,i] alpha[7]**MayMinTemp[y-71] +SpatRE[IDSpatial[i]]

    tau1[y,i]<-1/(a+b*log(dbh[y,i]))

  }
  }

  #priors
  cp~dunif(0,1.25)

  a~dlnorm(1,0.001)
  b~dnorm(0,0.001)

  for(i in 1:6){
    alpha[i]~dnorm(0,0.001)
   }

   ccomb<-c[1]+c[2]
   acomb<-a[1]+a[2]

     #spatial effects

     SpatRE[1:55]~spatial.exp(mu[1:55],lon[1:55],lat[1:55],tauS,phi,1)

    tauS~dgamma(0.001,0.001)
     phi~dunif(0.001,10)
     for(i in 1:55){  mu[i]<-0 }
}
```

Analysis of GNES:

```
model{
```

```
  for(i in 1:2051){

    #missign values
    MayMinTemp[i]~dnorm(0,1)

    GNEbaiS[i]~dnorm(B[i],tau1[Indv[i]])
    GNEbaiS.h[i]~dnorm(B[i],tau1[Indv[i]]) #predictions

    B[i]<- beta[Indv[i]]+lambda[Indv[i]]*CO2[i]+gamma[Indv[i]]*MayMinTemp[i]+SpatRE[IDSpatial[i]]#

  }


  #priors
  for(i in 2:55){
    beta[i]~dnorm(0,0.001)
    lambda[i]~dnorm(0,0.001)
    gamma[i]~dnorm(0,0.001)

    tau1[i]~dgamma(0.0001,0.0001)

   }

     SpatRE[1:55]~spatial.exp(mu[1:55],lon[1:55],lat[1:55],tauS,phi,1)

     tauS~dgamma(0.001,0.001)
     phi~dunif(0.001,10)
     for(i in 1:55){  mu[i]<-0 }
  }
```

Analysis of the slopes:

```
model{

  for(i in 2:NN){
  J[i]<-step(minerS[i]-cp)

  Ltau[i]<-1/(Lsd[i]*Lsd[i])

  Lambdamean[i]~dnorm(L [i],Ltau[i])
  Lambdamean.h[i]~dnorm(L[i],Ltau[i])
  L[i]<-(theta[1]+(theta[2]*J[i])+(theta[3]+theta[3]*J[i])*(minerS[i])
  }

  #priors
  for(i in 1:4){
    theta[i]~dnorm(0,0.001)
   }
   Thetacomb[1]<-theta[1]+theta[2]
   Thetacomb[2]<-theta[3]+theta[4]
   cp~dunif(0,1.25)

}
```

Supplementary Figures

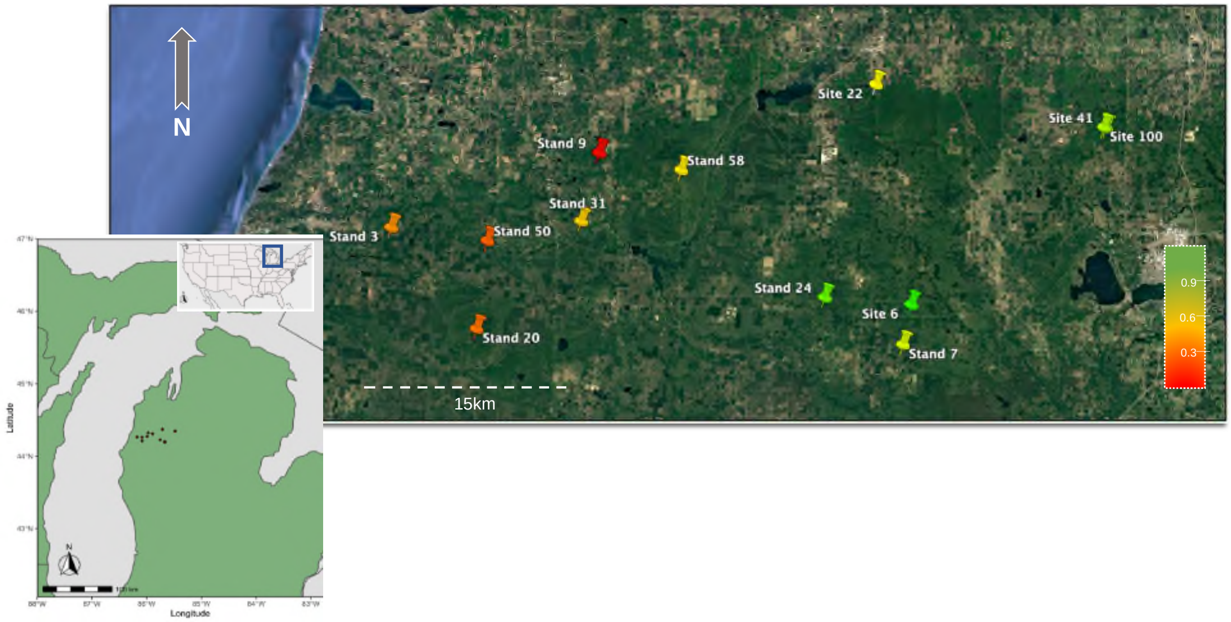

Supplementary Figure 1. Map of the twelve forest sites in Wexford and Manistee Counties, Manistee National Forest, Michigan, USA. All trees lay between 70-130m elevation. Pins are colored by rates of net N mineralization (µg g^{-1} d^{-1}). Satellite imagery May 2018. Insets: Michigan USA with sites in red; continental United States and location of Michigan, blue box. Study area is extensively described in[1,2]. Satellite imagery is derived from Google Earth.

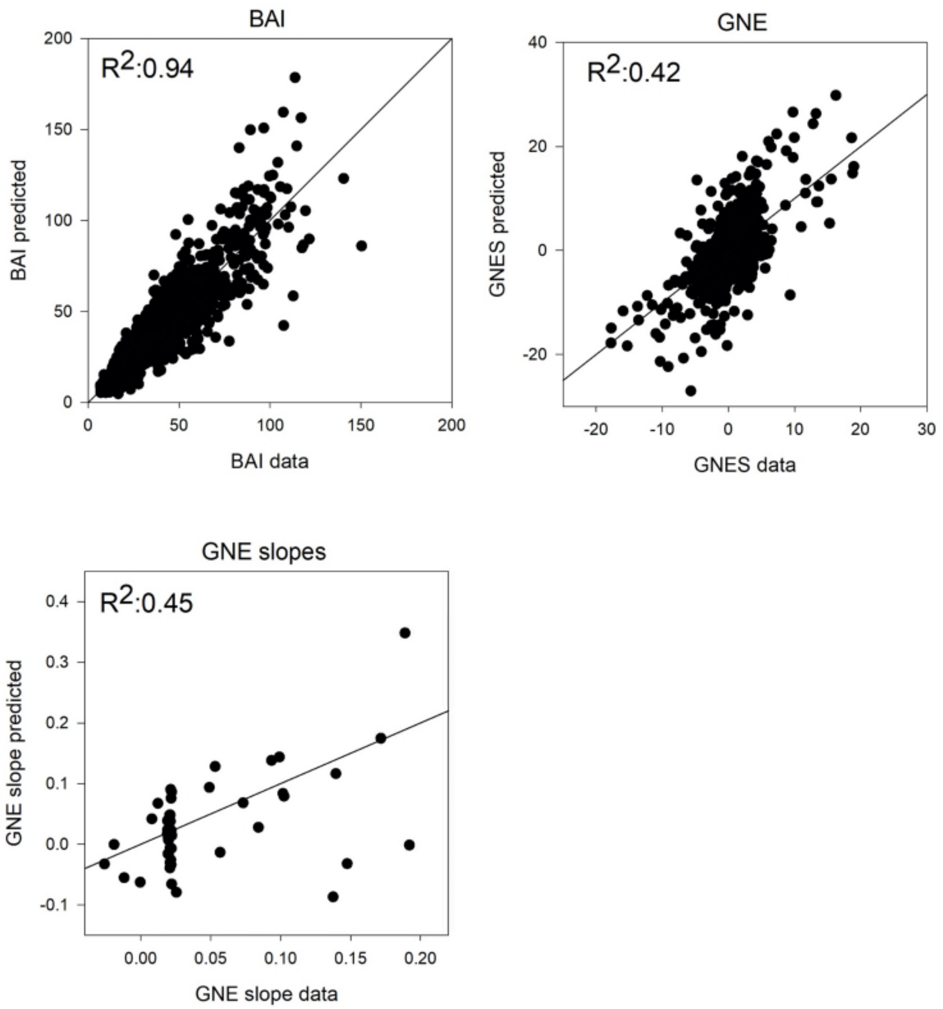

Supplementary Figure 2: Bayesian model fits, goodness of fit (predicted versus observed [in our case calculated data]) for our three dendrochronological analyses of plant growth. Solid line indicates the 1:1 relationship between the two variables. BAI = Basal Area Increment. GNES= standardized Growth Nitrogen Efficiency. See methods for calculation and estimations of parameters.

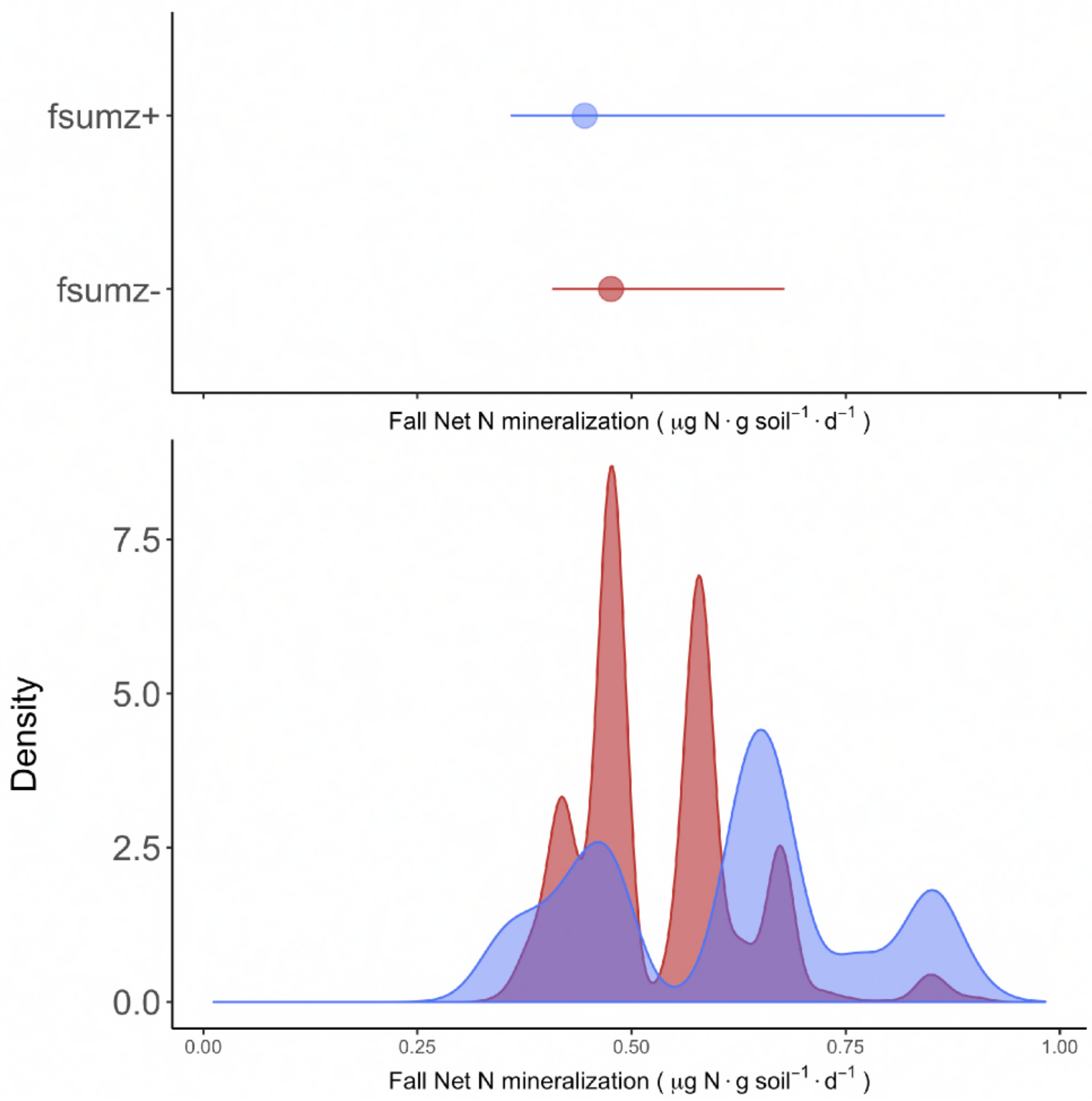

Supplementary Figure 3: Top panel, community change points where compositional change in ECM taxonomic membership is greatest. ECM taxa are grouped at the genus level. Blue indicates genera with (z+) scores, red indicates genera with (z-) scores. Community change point = 0.47µg g^{-1} d^{-1} (z+) and 0.45 µg g^{-1} d^{-1} (z-) scores. The observed (z+) and (z-) maxima are plotted as circles with the 95th percentile of their distributions as horizontal lines. The bottom panel shows the estimated probability densities across all booststrap replicates.

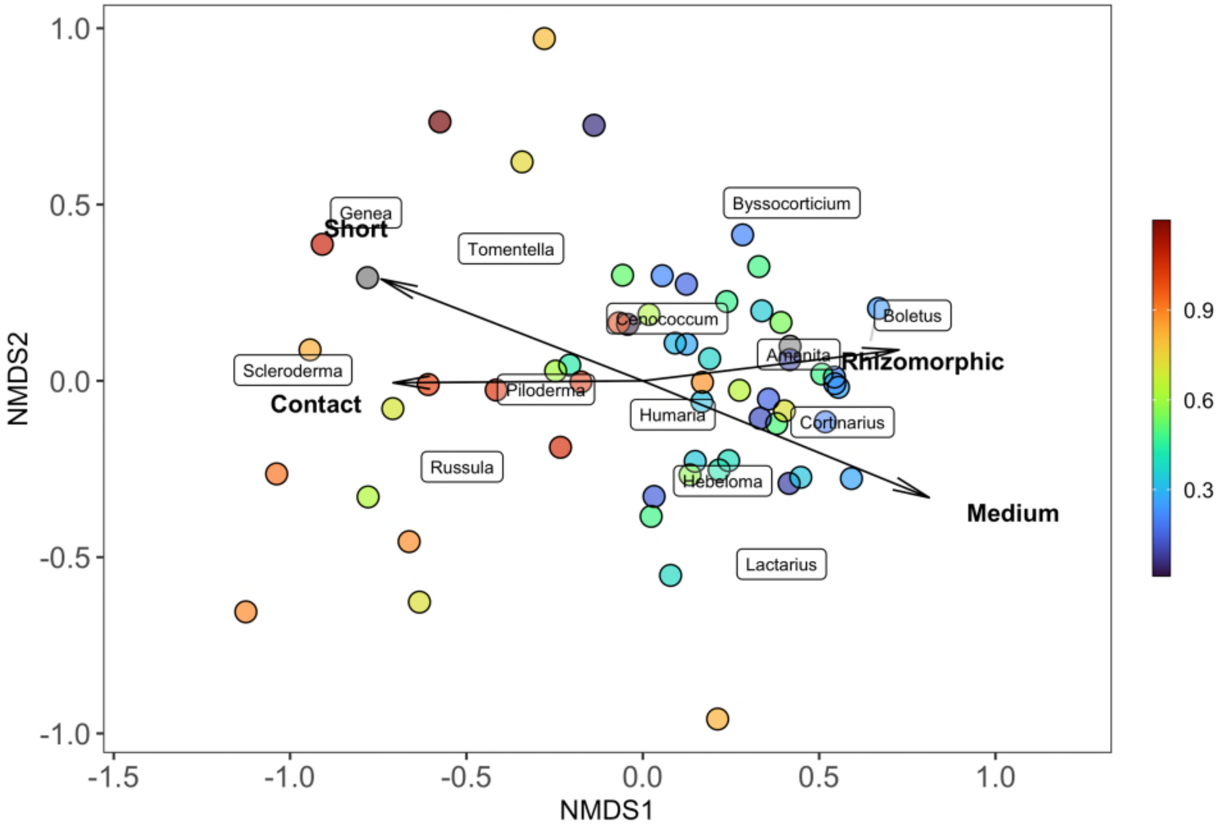

Supplementary Figure 4. NMDS plot of ECM fungal communities (points) colored by soil mineralization rates (µg N per gram dry soil per day; legend bar). The vectors indicate direction and degree of correlation between the two first NMDS axes and ECM fungal morphotypes. Medium and Short indicate hyphal exploration types and Contact and Rhizomorphic indicate rhizomorph presence. Location of plotted genus names are scaled centroid coordinates for abundant ECM fungal genera.

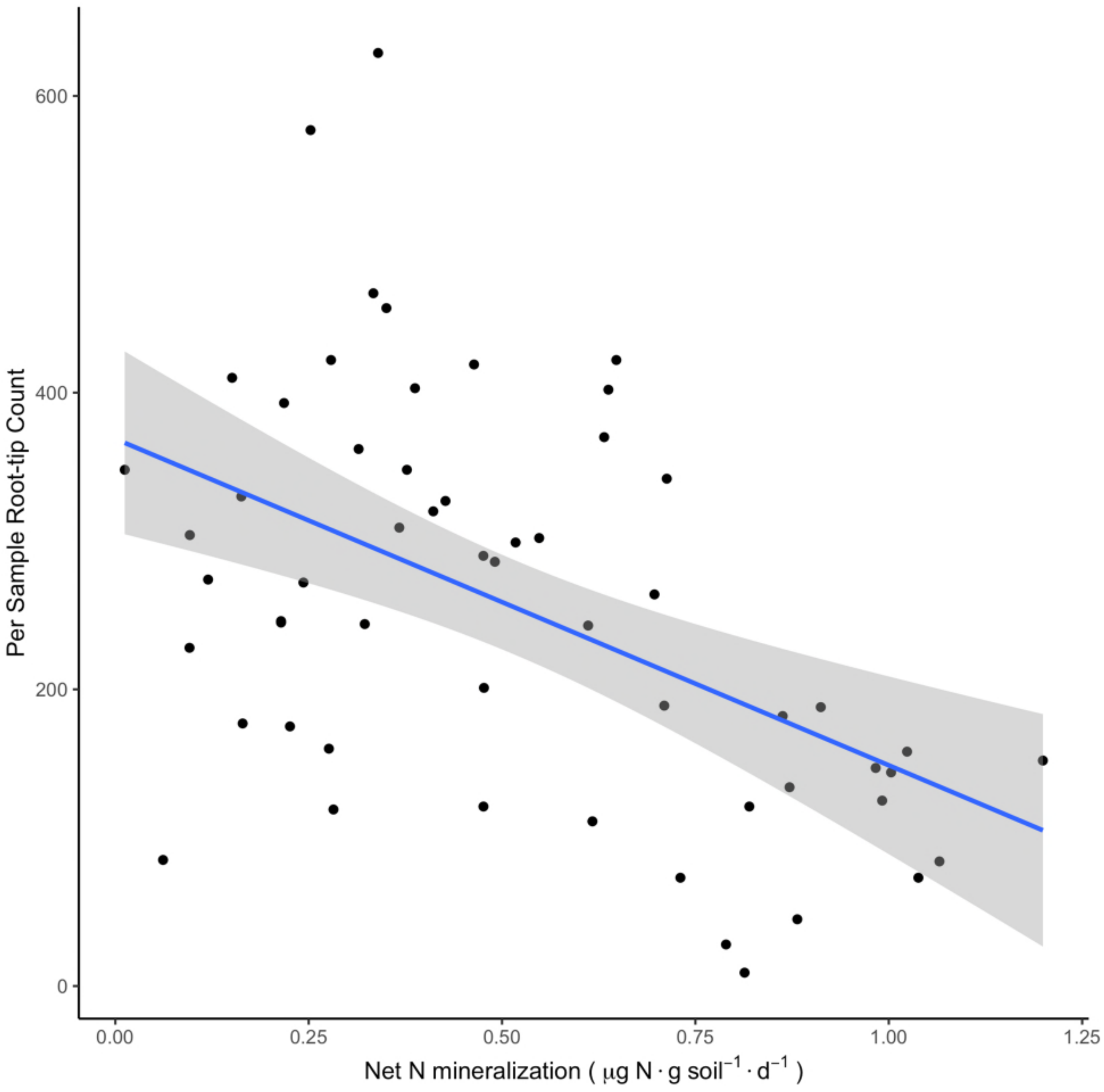

Supplementary Figure 5: Number of colonized ectomycorrhizal (ECM) fungal root-tips on northern red oak individuals across the studied soil gradient. Linear regression model: R^2_{adj} = 0.25, P =0.0001. Method = 'qr'. Coloured band indicates 95% confidence interval.

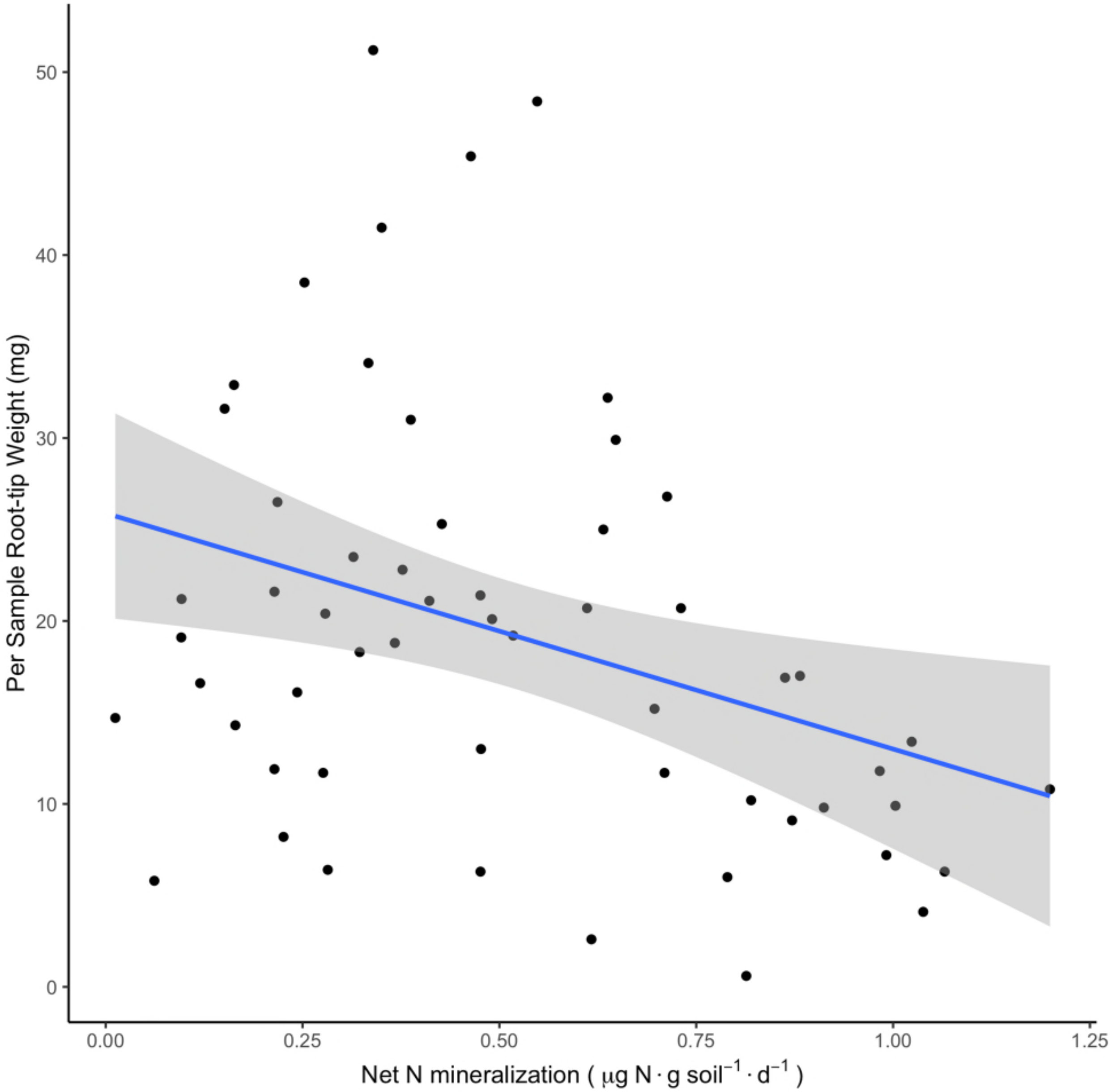

Supplementary Figure 6. Freeze-dried weight (mg) of ectomycorrhizal (ECM) fungal root-tips collected from northern red oak individuals across the studied inorganic N gradient: Linear regression model. Method = ‘qr’. R^2_{adj} = 0.10, P= 0.009. Coloured band indicates 95% confidence interval.

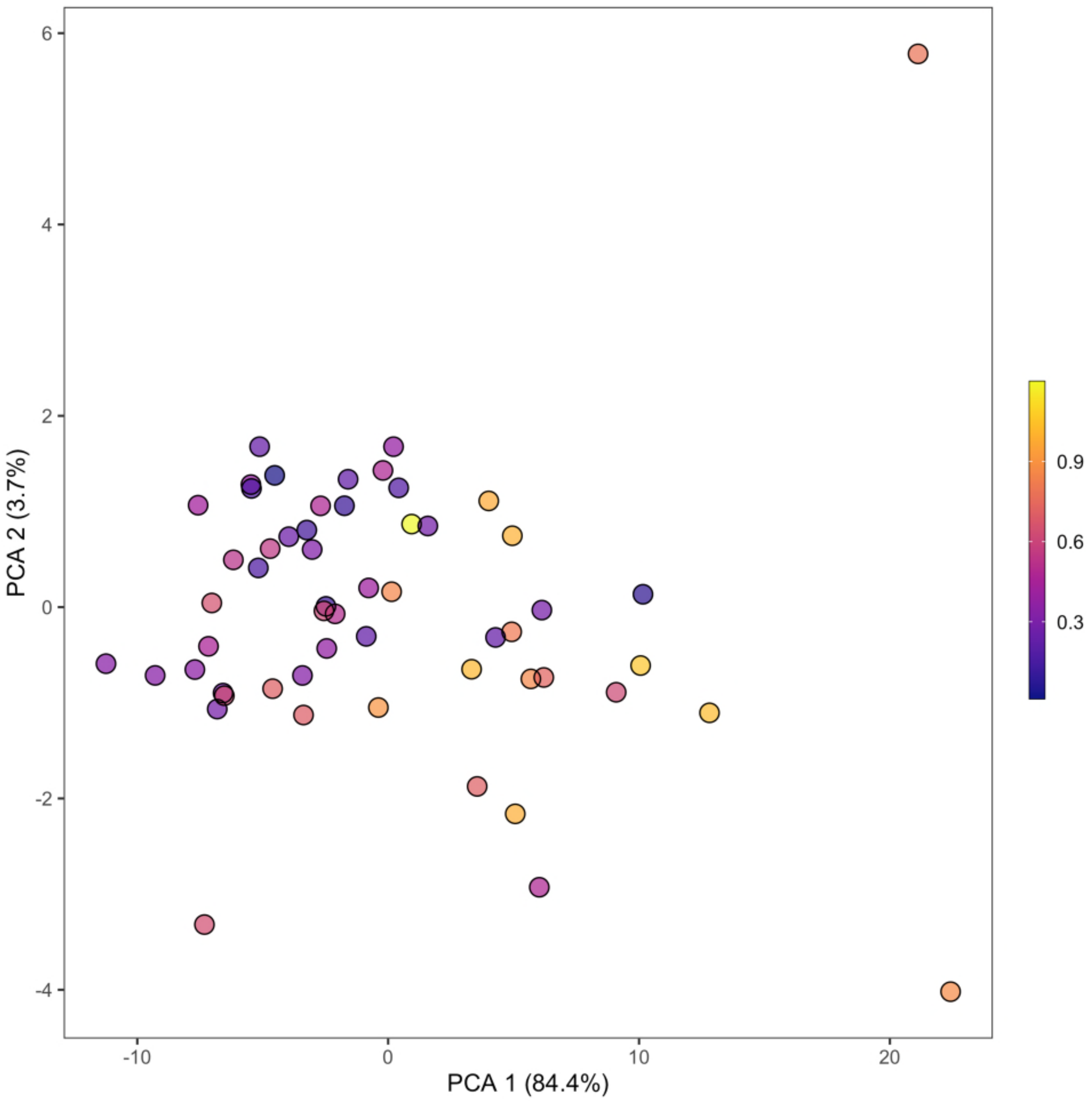

Supplementary Figure 7. Principal Component Analysis (PCA) for the abundances of the 100 decay genes studied here using shotgun metagenomic sequencing. Gene abundances were standardized using genes present at near single genome copies, and then weighted by the number of root-tips present in each sample and log-transformed. Points represent individual ECM communities colored by rates of net N mineralization (µg inorganic N g^{-1} d^{-1}).

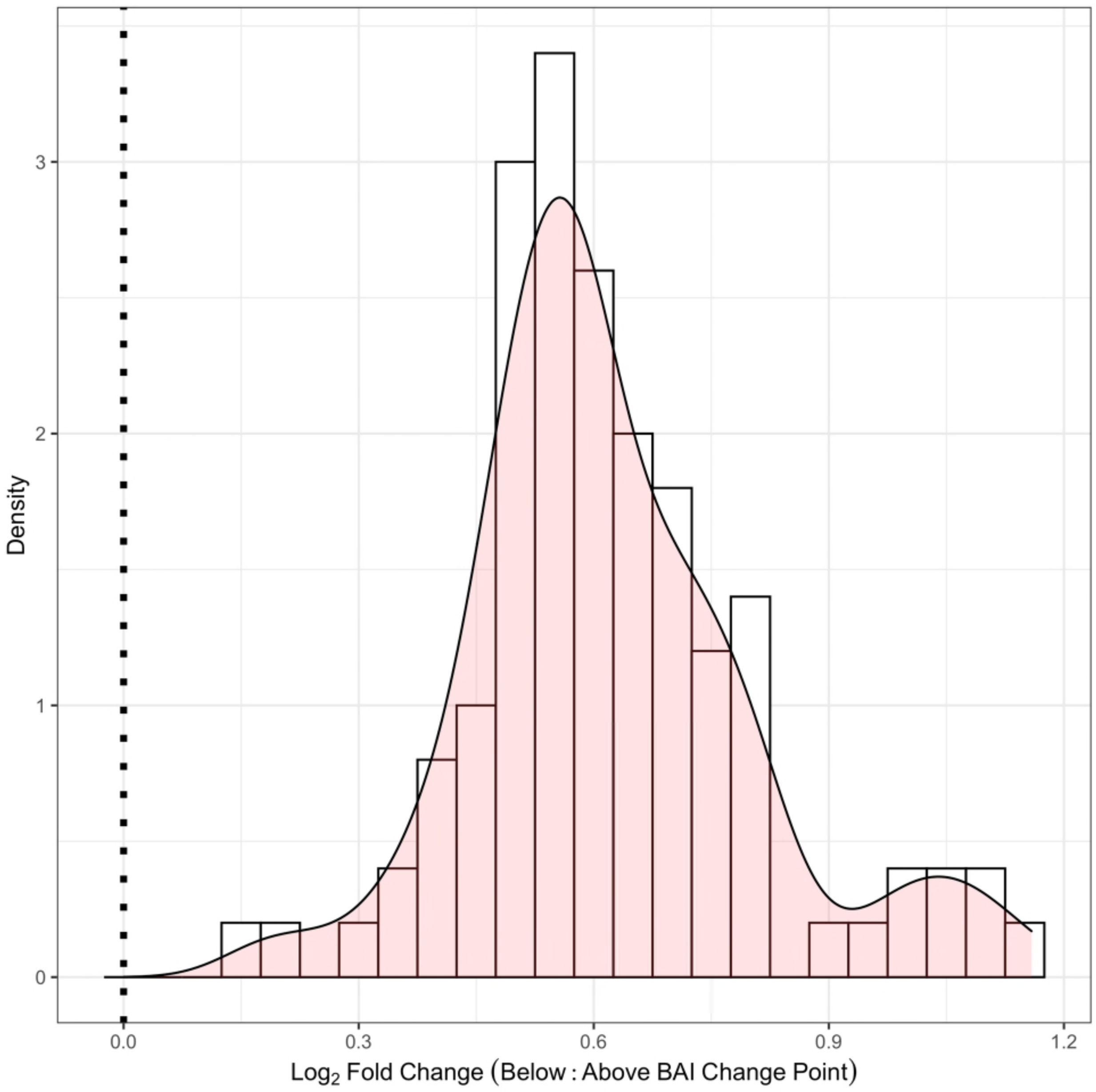

Supplementary Figure 8: Fold change values for all 100 gene families above and below the statistical BAI change point (0.53µg inorganic N g^{-1} d^{-1}). Each gene family was evaluated at a greater relative abundance below the statistical change point than above. Dashed line indicates equal gene counts above and below the change point.

A. B.

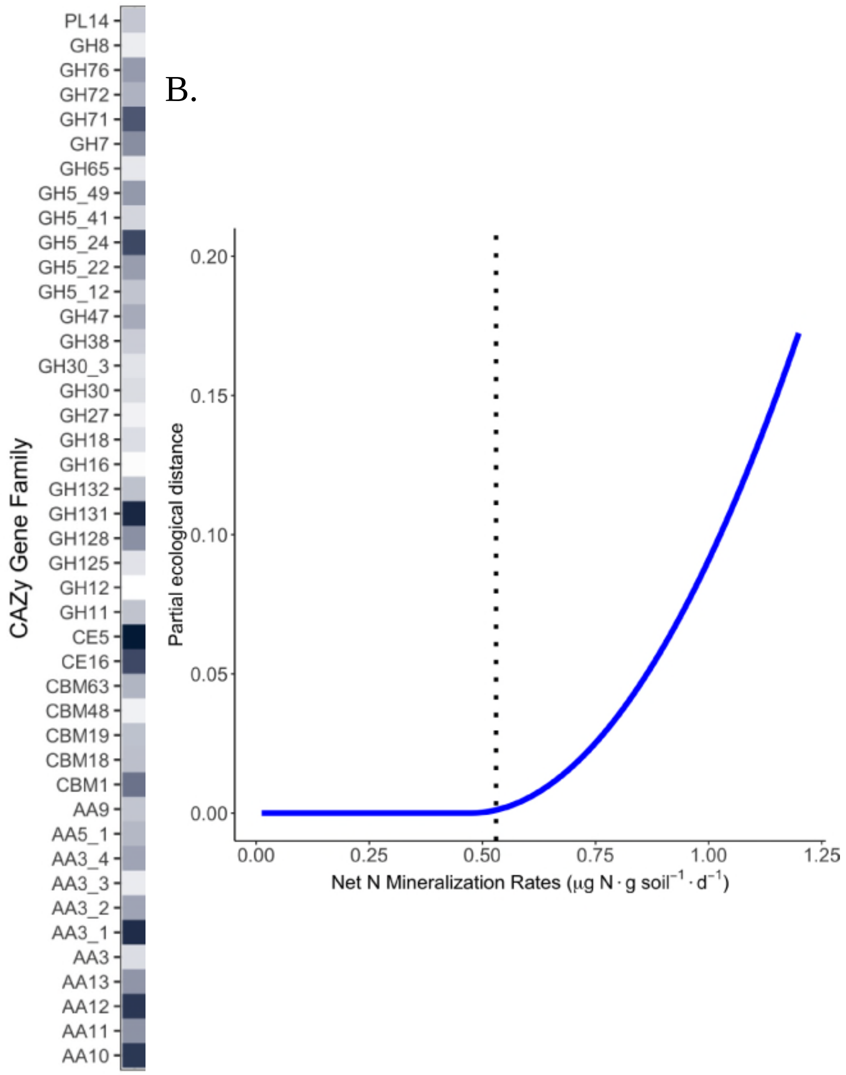

Supplementary Figure 10. A). Enrichment of (log_2 fold change) CAZy gene families below the statistical change point, relative to those occurring above the changepoint (basal area increment (BAI) change point). Gene families with greatest fold changes presented. Gene family names presented in Supplementary Table 2 with statistical information presented in Supplementary Table 3 **B).** Results from generalized dissimilarity model (GDM) documenting threshold response of ECM community aggregated decay potential for all 100 gene families studied here, measured as the partial ecological distance (y-axis) in response to empirical supply rates of inorganic N availability (Net N Mineralization). The slope of the blue line shows the rate of compositional metagenomic change along the soil gradient. Vertical dashed line denotes independently derived dendrochronological BAI changepoint (Figure 2; 0.53 µg inorganic N • g soil^{-1} • day $^{-1}$). The near-zero slope to the left of the vertical dashed line denotes negligible compositional change in the decay attributes of ECM communities along this portion of the soil gradient.

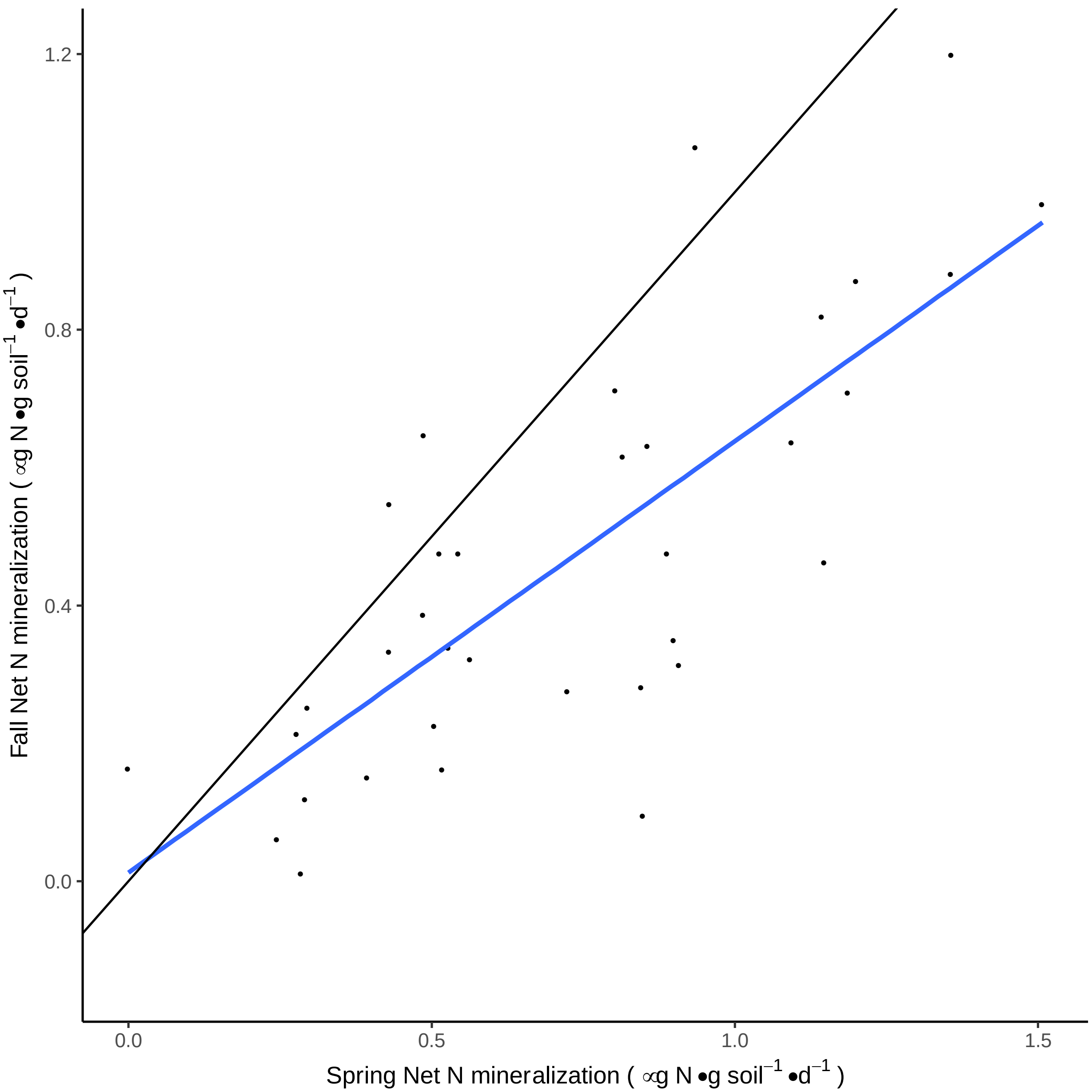

Supplementary Figure 11: Relationship between Spring and Fall mineralization rates for soils collected 2018. Paired samples are derived from the base of the same individual trees (Linear regression model: R^2_{adj}: 0.58. P <0.00001). Method = ‘qr’. Black line is 1:1 plot. Several samples were disturbed during the Spring 2018 incubation, accounting for the fewer total points plotted. Colored band indicates 95% confidence interval.

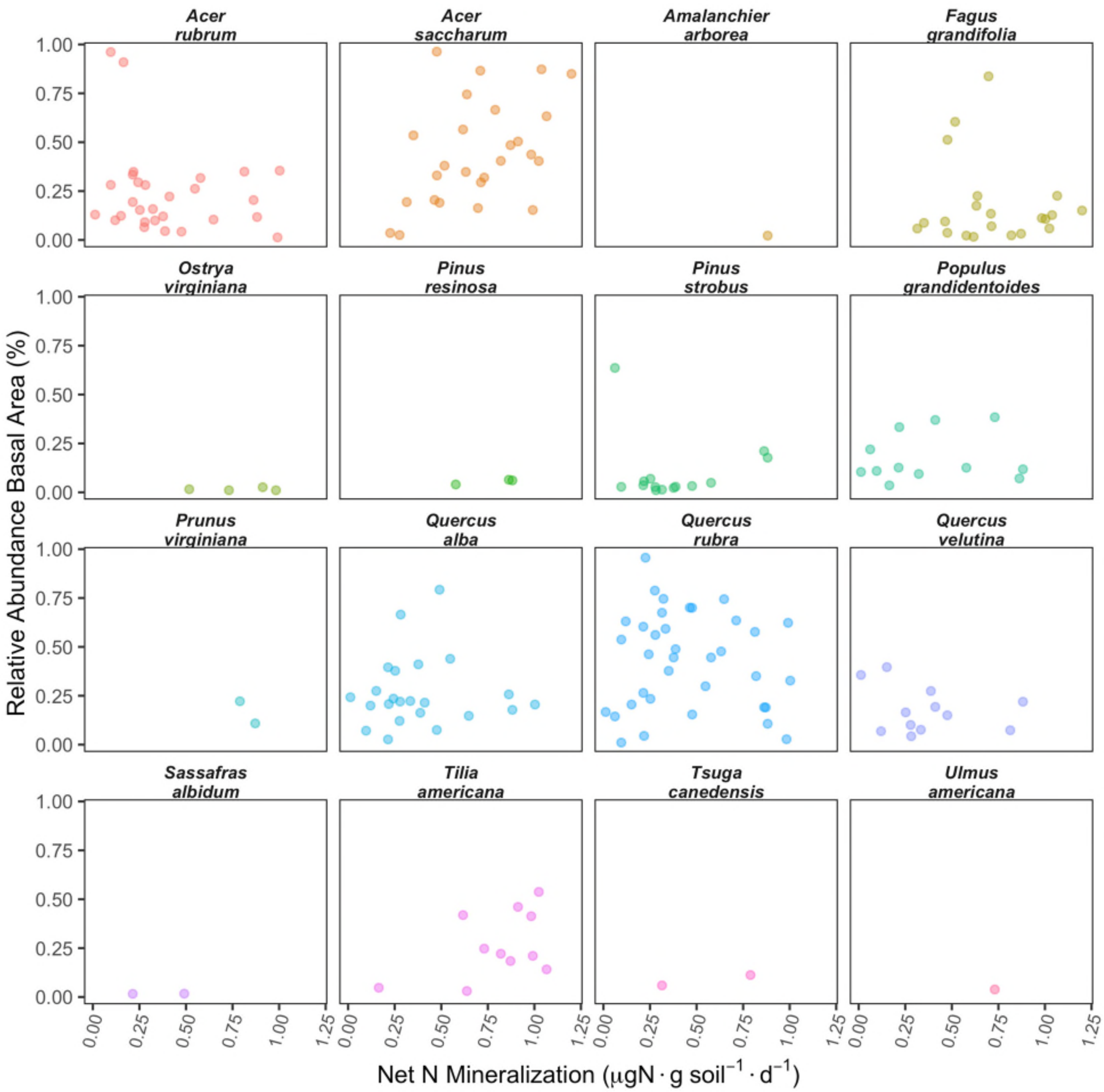

Supplementary Figure 12. Neighborhood overstory plant communities. Relative abundance of plant stems greater than 10cm within 10m radius of each focal *Quercus rubra* individual along the soil Net N mineralization gradient. Only stems at greater than 1% relative abundance shown.

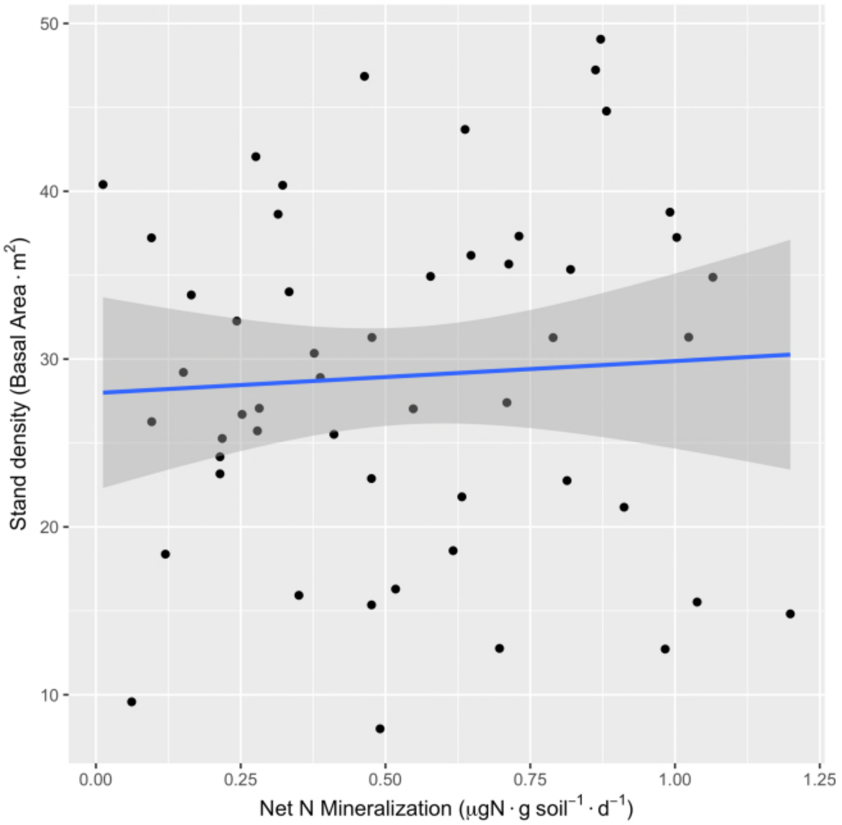

Supplementary Figure 13. Overstory stand density for the 'neighborhood' surrounding each focal *Quercus rubra* individual (i.e. within 10m radius). No significant differences along the soil mineralization gradient (P = 0.64). Linear regression model, method = 'qr'. Shaded band denotes 95% confidence interval.

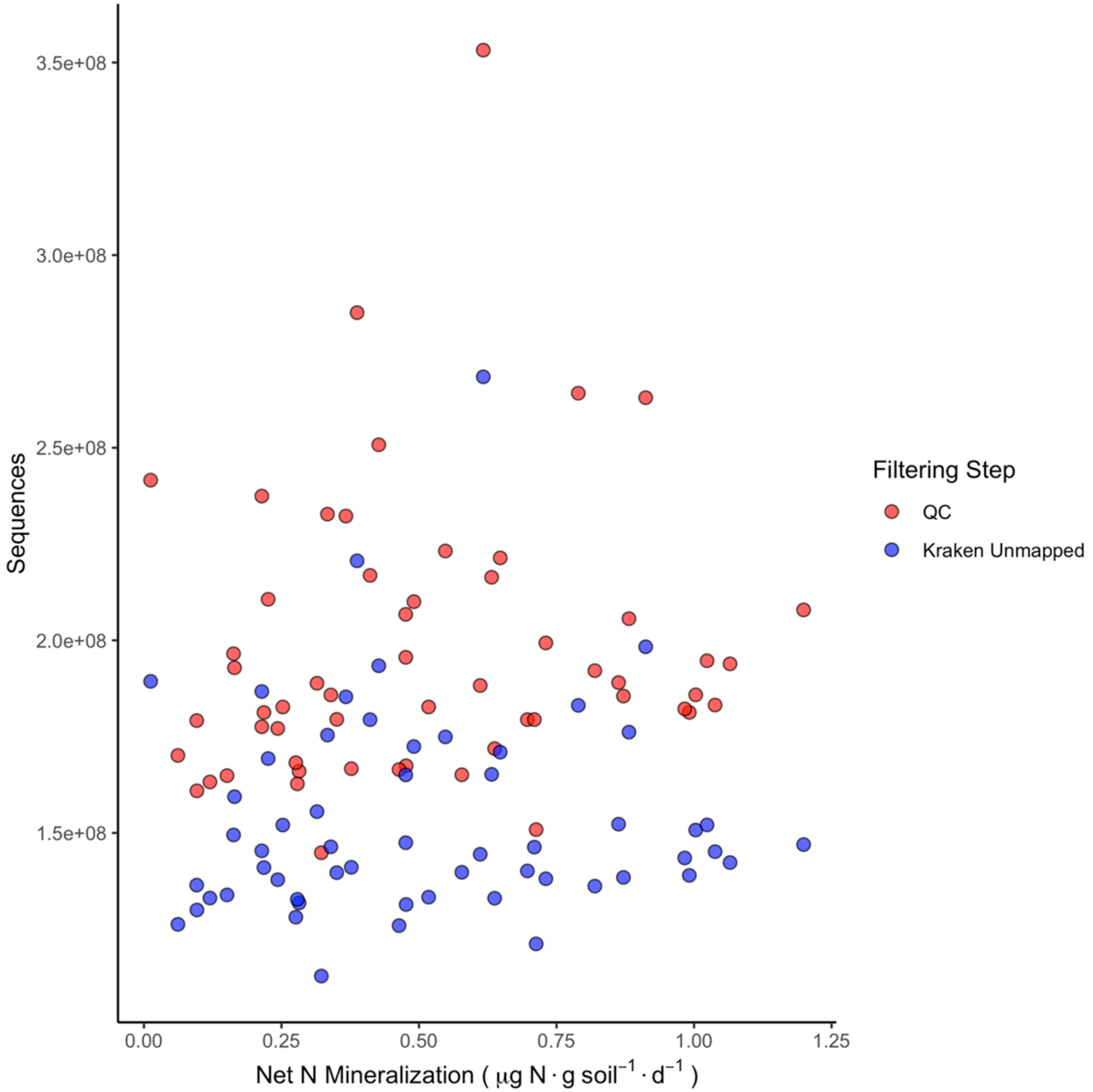

Supplementary Figure 14. Metagenomic sequencing yield for each sample. QC (red) represent quality filtered metagenomic reads, see main text. Kraken Unmapped (blue), represent reads that remain after Kraken filtering against plant and contaminant databases (putative fungal reads). No significant relationships across the soil gradient: linear regression: QC: P = 0.36. Kraken Unmapped: P= 0.73. Linear regression model, method = ‘qr’.

Supplementary Tables:

Supplementary Table 1: PCR primers employed in this study.

List of all primers used	
5.8S-Fun	AACTTTYRRCAAYGGATCWCT
ITS4-Fun	AGCCTCCGCTTATTGATATGCTTAART

Supplementary Table 2: Gene families, enzymes they encode and putative substrates. Sourced from (CAZy: http://www.cazy.org; http://peroxibase.toulouse.inra.fr/) and (CAZypedia.org)

Gene Family	Encoded Enzyme	Substrate
AA1	Laccases	Phenolics/lignin
AA10	Lytic polysaccharide monooxygenases	Cellulose/chitin
AA11	Lytic polysaccharide monooxygenases	Chitin
AA12	Oxidoreductase	Cellulose
AA13	Lytic polysaccharide monooxygenases	Starch
AA2	Peroxidases	Phenolics/lignin
AA3	GMC oxidoreductase	Cellulose/Lignin/Chitin
AA3_1	Cellobiose dehyrdogenase	Cellulose/Lignin/Chitin
AA3_2	Aryl Oxidases and glucose oxidase	Cellulose/Lignin/Chitin
AA3_3	Alcohol oxidase	Cellulose/Lignin/Chitin
AA3_4	Pyranose Oxidase	Cellulose/Lignin/Chitin
AA5	Copper radical oxidases	Lignin and intemediaries
AA5_1	Glyoxal oxidase	Lignin and intemediaries
AA5_2	Galactose oxidaase	Lignin and intemediaries
AA6	Benzoquinone reductase	Aromatics
AA8	Iron Reductase	Unknown
AA9	Lytic polysaccharide monooxygenases	Cellulose
CBM1	NA	Cellulose
CBM12	NA	Cellulose
CBM13	NA	Cellulose
CBM18	NA	Cellulose
CBM19	NA	Cellulose
CBM21	NA	Cellulose
CBM43	NA	Cellulose

CBM48	NA	Cellulose
CBM52	NA	Cellulose
CBM63	NA	Cellulose
CE1	Carbohydrate esterases	Varied
CE12	Carbohydrate esterases	Unknown
CE16	Acetylesterase	Unknown
CE4	Carbohydrate esterases	Acetyl groups in polysaccharides
CE5	Acetyl Xylan esterase & cutinases	Varied
CE8	Pectin methylesterase	Pectin
CE9	Carbohydrate esterases	Acetyl groups in polysaccharides
GH1	β-glucosidase	Varied
GH10	Endo-1,4-β- xylanase	Cellulose/Hemicellulose
GH11	Endo-1,4-β-xylanase	Xylan/Cellulose/Hemicellulose
GH114	Endo-α-±-1,4 polygalactosaminidase	Polygactoseamine
GH115	xylan-a-1-2-glucoronidase	Xylan
GH12	Endo- β -1,4-glucanase	xyloglucan
GH125	exo-α-1-6-mannosidase	mannose sugers
GH128	Glycoside hydrolase	Varied
GH13	1,4-α-Glucan-branching enzyme	Varied
GH131	exo-β-glucanase	Cellulose
GH132	β-1-3-glucan	Cellulose
GH133	amlyo-α-1-6-glucosidase	Glycogen
GH15	glucoamylase	Varied
GH16	β-1,3-Glucanase	Glucans/Galactans
GH17	Glucan-endohydrolases	Glucans
GH18	Chitinase	Chitin
GH19	Chitinase	Chitin
GH2	β-galactosidases	Galactose
GH20	β-hexosaminidase	Chitin
GH23	peptidoglycan lyases	Peptidoglycan
GH26	Endo-β-1,4 mannases	Mannose sugers
GH27	α-galactosidase	Varied
GH28	polygalacturonidase	Galactose
GH29	exo-α-fucosidases	Fucose
GH3	β-glucosidase	Cellulose
GH30	endo-β-1-4-xylanase	Xylan
GH30_3	endo-β-1-4-xylanase	Xylan

GH31	α-glucosidase	Xylan
GH35	β-galactosidases	Galactose
GH37	α-trehalase	Trehalose
GH38	α-mannosidase	Mannose sugers
GH43	β-xylosidase	Arabinose
GH46	chitosinase	Chitin
GH47	α-mannosidase	Mannose sugers
GH5	Endo-β-1,4-glucanase	Cellulose/varied
GH5_12	Glycoside hydrolase	Cellulose/varied
GH5_22	Glycoside hydrolase	Cellulose/varied
GH5_24	Glycoside hydrolase	Cellulose/varied
GH5_41	Glycoside hydrolase	Cellulose/varied
GH5_49	Glycoside hydrolase	Cellulose/varied
GH5_5	Endo-β-1,4-glucanase	Cellulose/varied
GH5_7	Endo-1,4-β-mannanase	Cellulose/varied
GH5_9	endoclucanase	Cellulose/varied
GH51	endoclucanase	Hemicellulose
GH53	Endo-1,4-β-galactanase	Hemicellulose
GH6	Cellobiohydrolase 2	Cellulose
GH62	α-L-arabinofuranosidase	Xylan
GH63	α-glucosidase	Varied
GH65	α-trehalase	Varied
GH7	Cellobiohydrolase 1	Cellulose/varied
GH71	α-1-3-gluconase	Cellulose/varied
GH72	β-1,3-glucanosyltransglycosylase	Varied
GH74	endoglucanase	Glucans
GH76	α-1,6-mannanas	Mannose sugers
GH77	amylomaltase	Varied
GH78	α-L-rhamnosidase	Varied
GH8	chitosinase	Varied
GH81	endo-β-1,3-glucanase	Glucans
GH85	endo-β-N-acetylglucosaminidase	Varied
GH88	β-glucuronyl hydrolase	Glucans
GH9	Glycoside hydrolase	Cellulose
GH95	α-L-fucosidase	Varied
DyPrx	Dye-decoloring peroxidase	Lignin and intemediaries
LiP	Lignin peroxidase	Lignin and intemediaries
MnP	Manganese peroxidase	Lignin and intemediaries

Supplementary Table 3. Results of one-way ANOVA (1-sided) for the relative abundance of CAZy gene families above and below the basal area increment (BAI) change point. P values are Bonferroni corrected.

Gene ID	SS	MS	statistic	p.value	p.adjust
AA1	1358.285	1358.285	9.837	0.003	0.206
AA10	26.058	26.058	13.862	0.000	0.040
AA11	1221.870	1221.870	13.025	0.001	0.056
AA12	1370.698	1370.698	16.511	0.000	0.015
AA13	62.767	62.767	6.160	0.016	0.557
AA2	245.721	245.721	4.213	0.045	0.720
AA3	33027.744	33027.744	8.549	0.005	0.323
AA3_1	591.208	591.208	9.559	0.003	0.217
AA3_2	10085.599	10085.599	11.747	0.001	0.095
AA3_3	61977.388	61977.388	6.435	0.014	0.557
AA3_4	2891.849	2891.849	2.390	0.128	0.987
AA5	1872.023	1872.023	2.996	0.089	0.981
AA5_1	1181103.298	1181103.298	9.954	0.003	0.199
AA5_2	395.009	395.009	3.917	0.053	0.741
AA6	1375.186	1375.186	6.895	0.011	0.516
AA8	2.039	2.039	2.825	0.099	0.987
AA9	223.837	223.837	13.122	0.001	0.054
CBM1	635.321	635.321	18.337	0.000	0.007
CBM12	134.078	134.078	5.389	0.024	0.602
CBM13	185.945	185.945	5.860	0.019	0.568
CBM18	634.917	634.917	14.707	0.000	0.029
CBM19	551.007	551.007	6.510	0.014	0.557
CBM21	308.888	308.888	2.763	0.102	0.987
CBM43	427.682	427.682	9.829	0.003	0.206
CBM48	111.285	111.285	7.661	0.008	0.440
CBM52	0.931	0.931	0.188	0.666	0.987

CBM63	112.566	112.566	9.726	0.003	0.206
CE1	75.128	75.128	6.866	0.011	0.516
CE12	68.292	68.292	5.598	0.022	0.590
CE16	760.567	760.567	21.003	0.000	0.003
CE4	32.315	32.315	6.303	0.015	0.557
CE5	131.973	131.973	15.666	0.000	0.021
CE8	273.160	273.160	6.485	0.014	0.557
CE9	33.797	33.797	7.099	0.010	0.502
GH1	105.911	105.911	8.180	0.006	0.364
GH10	76.089	76.089	6.499	0.014	0.557
GH11	21.731	21.731	12.816	0.001	0.060
GH114	27.153	27.153	4.565	0.037	0.670
GH115	271.284	271.284	6.893	0.011	0.516
GH12	79.783	79.783	9.115	0.004	0.258
GH125	134.077	134.077	9.121	0.004	0.258
GH128	1146.442	1146.442	15.320	0.000	0.024
GH13	127.699	127.699	5.859	0.019	0.568
GH131	703.951	703.951	20.494	0.000	0.003
GH132	33.794	33.794	15.522	0.000	0.022
GH133	1599.886	1599.886	7.119	0.010	0.502
GH15	532.407	532.407	5.587	0.022	0.590
GH16	167.570	167.570	9.855	0.003	0.206
GH17	254.061	254.061	8.747	0.005	0.299
GH18	69.683	69.683	11.722	0.001	0.095
GH19	15.466	15.466	4.940	0.030	0.670
GH2	194.834	194.834	5.645	0.021	0.590
GH20	89.060	89.060	6.094	0.017	0.557
GH23	20.100	20.100	6.493	0.014	0.557
GH26	31.401	31.401	4.153	0.046	0.720
GH27	2341.262	2341.262	8.194	0.006	0.364
GH28	276.378	276.378	6.179	0.016	0.557
GH29	318.314	318.314	4.682	0.035	0.670
GH3	272.957	272.957	7.422	0.009	0.468
GH30	46.522	46.522	9.838	0.003	0.206
GH30_3	2166.922	2166.922	7.606	0.008	0.444
GH31	216.331	216.331	7.167	0.010	0.501
GH35	569.398	569.398	7.401	0.009	0.468
GH37	83.100	83.100	7.751	0.007	0.428
GH38	278.789	278.789	11.104	0.002	0.122

GH43	30.377	30.377	5.919	0.018	0.568
GH46	1.565	1.565	0.983	0.326	0.987
GH47	2931.383	2931.383	14.396	0.000	0.033
GH5	58.800	58.800	6.558	0.013	0.557
GH5_12	4167.814	4167.814	11.419	0.001	0.107
GH5_22	5873.513	5873.513	10.467	0.002	0.160
GH5_24	10470.868	10470.868	14.355	0.000	0.033
GH5_41	14.526	14.526	9.746	0.003	0.206
GH5_49	51.486	51.486	14.772	0.000	0.029
GH5_5	7.782	7.782	5.306	0.025	0.603
GH5_7	212.097	212.097	4.516	0.038	0.670
GH5_9	1359.486	1359.486	9.351	0.003	0.236
GH51	145.282	145.282	3.406	0.070	0.845
GH53	30.912	30.912	2.758	0.103	0.987
GH6	49.866	49.866	6.605	0.013	0.557
GH62	5.780	5.780	1.941	0.169	0.987
GH63	98.263	98.263	8.128	0.006	0.364
GH65	130.154	130.154	8.297	0.006	0.358
GH7	8.697	8.697	14.815	0.000	0.028
GH71	3044.962	3044.962	18.561	0.000	0.007
GH72	249.726	249.726	15.202	0.000	0.024
GH74	1079.441	1079.441	4.874	0.032	0.670
GH76	219.763	219.763	16.062	0.000	0.018
GH77	108.550	108.550	6.310	0.015	0.557
GH78	850.479	850.479	5.285	0.025	0.603
GH8	30.159	30.159	7.545	0.008	0.449
GH81	107.906	107.906	7.089	0.010	0.502
GH85	10.821	10.821	3.661	0.061	0.793
GH88	52.006	52.006	7.392	0.009	0.468
GH9	165.914	165.914	6.967	0.011	0.509
GH95	536.530	536.530	4.779	0.033	0.670
PL14	549.135	549.135	8.262	0.006	0.358
DyPrx	5526.286	5526.286	2.166	0.147	0.987
LiP	2001.969	2001.969	1.399	0.242	0.987
MnP	1851.988	1851.988	2.569	0.115	0.987

Supplementary Table 4: Generalized Dissimilarity Model (GDM) output: Variables contribute to differentiation of fungal community decay (metagenomic) potential for the gene families studied here. Backwards model selection initially procedure initially included all environmental

variables described in the main-text and geographic distance between plots. Reduced models are presented below after backward variable elimination with 500 permutations.

[1] Model Selection Summary	
	Full Model
Model deviance	205.75
Percent deviance explained	13.24
Model p-value	0.0749
Fitted permutations	494
[2] Variable Importance (% of unique variance attributed to each variable)	
	Full Model
Geographic	3.164
C	10.825
C:N	14.643
Mineralization rates	20.042
[3] Variable Significance (P-value)	
	Full Model
Geographic	0.0692
C	0.244
C:N	0.192
Mineralization rates	0.162
[4] Number of permutations used to calculate model statistics	
	Full Model
Geographic	491
C	500
C:N	500
Mineralization rates	500

Supplementary Table 5: Analysis of BAI, parameter posterior means, SD, and 95%CI. In bold: coefficients that are statistically significant (95%CI does not include zero).

Parameter	mean	SD	2.5%	97.5%
$\alpha 1$ intercept before the change point	5.18	0.46	4.2	5.87
$\alpha 2$ difference in intercept	-1.49	0.22	-1.91	-1.03
$(\alpha 2+\alpha 3)$ intercept after the change	3.93	0.25	3.45	4.33
$\alpha 3$ N slope before change point	-0.41	0.37	-1.34	0.27

$\alpha4$ difference in N slope	1.99	0.32	1.39	2.65
$(\alpha3+\alpha4)$ N slope after change point	**1.89**	**0.27**	**1.29**	**2.36**
$\alpha5$ ln(dbh)	**0.41**	**0.04**	**0.33**	**0.49**
$\alpha6$ BAIS	**0.26**	**0.01**	**0.24**	**0.29**
$\alpha7$ May tempS	**0.049**	**0.005**	**0.038**	**0.061**
Change point	0.53	0.01	0.51	0.55
a intercept var	0.039	0.009	0.001	0.05
b slope var	0.0036	0.002	0.0001	0.01
$1/\sigma^2$ SERE	0.016	0.005	0.004	0.02
φ	7.54	2.05	2.38	9.93

Supplementary Table 6: Analysis of GNES results, parameter posterior means, SD, and 95%CI.

intercept	mean	sd	2.50%	97.50%
$\beta1$	-68.09	38.08	-128.8	9.796
$\beta2$	-19.21	38.97	-93.21	79.94
$\beta3$	1.594	21.21	-38.3	44.14
$\beta4$	-25.32	19.98	-65.94	19.65
$\beta5$	28.74	18.88	-8.375	66.93
$\beta6$	45.6	20.17	10.21	87.9
$\beta7$	35.52	18.43	-4.103	66.92
$\beta8$	30.92	31.99	-20.27	112.6
$\beta9$	-1.769	20.71	-39.86	32.95
$\beta10$	32.46	15.86	3.119	62.18
$\beta11$	-34.88	25.62	-80.1	14.45
$\beta12$	33.51	12.28	13.66	58.58
$\beta13$	15	14.29	-8.499	49.26
$\beta14$	10.19	24.09	-36.8	46.55
$\beta15$	-46.85	9.916	-65.46	-28.07
$\beta16$	1.694	14.31	-28.29	28.19
$\beta17$	-7.824	24.75	-61.44	26.75
$\beta18$	2.16	13.38	-26.32	23.65
$\beta19$	-19.79	27.8	-54.93	52.18
$\beta20$	-9.799	22.3	-52.77	26.22
$\beta21$	20.22	15.21	-6.334	50.89
$\beta22$	34.71	25.8	-32.44	75.9
$\beta23$	-28.03	38.43	-149	10.22

β24	6.319	12	-15.98	29.61
β25	19.67	20.91	-20.98	49.31
β26	10.58	17.39	-17.31	40.83
β27	40.48	34.59	-1.412	122.5
β28	7.262	10.42	-10.2	29.5
β29	3.59	11.11	-17.51	24.21
β30	-32.56	17.06	-60.12	-6.484
β31	0.6273	21.33	-41.12	34.68
β32	-4.164	25.32	-35.31	50.47
β33	80.68	31.06	39.64	173.6
β34	17.78	5.31	7.268	29.02
β35	17.94	12.2	-1.233	42.31
β36	2.78	11.21	-20.47	24.29
β37	8.782	8.996	-7.088	23.75
β38	24.83	10.3	1.161	46.13
β39	-30.91	8.504	-46.14	-14.42
β40	-28.93	17.81	-65.68	-8.666
β41	-2.756	9.427	-16.62	15.14
β42	-21.3	8.039	-35.95	-5.94
β43	-15.47	9.361	-34.8	0.6734
β44	-11.74	10.72	-35.6	5.025
β45	-24.33	19.86	-54.12	14
β46	-4.467	9.55	-17.45	19.12
β47	14.31	5.536	1.949	23.93
β48	8.874	8.073	-5.686	23.4
β49	-8.608	6.984	-22.02	5.232
β50	-20.73	7.032	-36.44	-7.996
β51	14.79	23.8	-20.86	42.88
β52	7.208	7.606	-14.19	17.18
β53	-19.64	6.18	-27.34	-7.952
β54	-21.9	9.918	-37.46	-5.842
Slope CO_2	mean	sd	2.50%	97.50%
λ1	0.5435	0.09428	0.3511	0.7301
λ2	0.3723	0.05083	0.2884	0.4989
λ3	0.002042	0.06728	-0.1354	0.1305
λ4	0.1772	0.06134	0.0244	0.2753
λ5	0.007193	0.05524	-0.1005	0.1279
λ6	-0.07401	0.05254	-0.2065	0.01906

$\lambda 7$	0.1141	0.0443	0.0309	0.2022
$\lambda 8$	0.09569	0.04039	0.02564	0.1732
$\lambda 9$	0.09027	0.03823	0.00472	0.1609
$\lambda 10$	0.1422	0.03813	0.06587	0.2196
$\lambda 11$	0.1224	0.03531	0.04955	0.1929
$\lambda 12$	0.04161	0.03242	-0.01705	0.109
$\lambda 13$	0.06939	0.0298	0.0125	0.1279
$\lambda 14$	-0.01305	0.03291	-0.07098	0.04833
$\lambda 15$	0.1166	0.01613	0.08703	0.1466
$\lambda 16$	0.1029	0.02258	0.06292	0.154
$\lambda 17$	-0.07477	0.02628	-0.1222	-0.02035
$\lambda 18$	0.06242	0.02137	0.01755	0.1018
$\lambda 19$	0.0529	0.01996	0.01772	0.09554
$\lambda 20$	0.0428	0.02411	0.00164	0.09267
$\lambda 21$	-0.07074	0.02435	-0.1261	-0.02609
$\lambda 22$	-0.06218	0.01993	-0.1036	-0.02859
$\lambda 23$	-0.01869	0.02122	-0.06621	0.0201
$\lambda 24$	-0.04334	0.0277	-0.1041	0.006806
$\lambda 25$	0.09106	0.01894	0.05487	0.1261
$\lambda 26$	0.006957	0.02098	-0.03594	0.05271
$\lambda 27$	-0.0693	0.01844	-0.1022	-0.03357
$\lambda 28$	-0.04039	0.01872	-0.07592	-0.004332
$\lambda 29$	-0.03137	0.01961	-0.0704	0.002341
$\lambda 30$	0.09203	0.01109	0.06905	0.114
$\lambda 31$	0.01048	0.01632	-0.02155	0.04323
$\lambda 32$	0.01492	0.01481	-0.01495	0.04272
$\lambda 33$	-0.02795	0.01517	-0.05608	0.003311
$\lambda 34$	0.04278	0.01157	0.02171	0.06459
$\lambda 35$	-0.03646	0.01396	-0.06374	-0.009739
$\lambda 36$	-0.009294	0.01423	-0.03448	0.02071
$\lambda 37$	-0.03405	0.01265	-0.0575	-0.008858
$\lambda 38$	0.03896	0.01059	0.02015	0.06285
$\lambda 39$	0.04333	0.01021	0.01839	0.06197
$\lambda 40$	0.04674	0.00848	0.03157	0.0649
$\lambda 41$	-0.01905	0.01183	-0.04602	0.00418
$\lambda 42$	0.01606	0.0108	-0.003268	0.03581
$\lambda 43$	-0.005389	0.01241	-0.037	0.01736

$\lambda 44$	0.008525	0.01152	-0.01749	0.02787
$\lambda 45$	0.02317	0.009017	0.005742	0.04095
$\lambda 46$	0.01671	0.01093	-0.007164	0.0363
$\lambda 47$	0.01996	0.009079	0.001915	0.03866
$\lambda 48$	0.02079	0.009693	2.55E-05	0.0387
$\lambda 49$	-0.01889	0.009926	-0.04184	-0.001763
$\lambda 50$	0.008982	0.009556	-0.0108	0.02842
$\lambda 51$	0.01612	0.007141	0.001553	0.02963
$\lambda 52$	0.0251	0.006909	0.0118	0.03994
$\lambda 53$	0.04023	0.00468	0.03115	0.0496
$\lambda 54$	0.01659	0.007716	0.00236	0.03121
Slope May-tempS	mean	sd	2.50%	97.50%
$\gamma 1$	2.229	1.635	-0.8534	5.558
$\gamma 2$	2.258	1.109	0.03533	4.371
$\gamma 3$	4.753	1.687	1.484	8.096
$\gamma 4$	-0.3649	1.34	-2.955	2.296
$\gamma 5$	-1.652	1.199	-4.058	0.6895
$\gamma 6$	1.759	1.115	-0.4506	3.945
$\gamma 7$	2.073	0.9615	0.1877	3.966
$\gamma 8$	0.6324	0.7873	-0.9356	2.197
$\gamma 9$	1.004	0.7491	-0.4485	2.522
$\gamma 10$	0.8918	0.6683	-0.4314	2.2
$\gamma 11$	0.879	0.6239	-0.3428	2.113
$\gamma 12$	1.073	0.7208	-0.3655	2.473
$\gamma 13$	0.1184	0.6725	-1.22	1.43
$\gamma 14$	1.384	0.6344	0.1206	2.61
$\gamma 15$	0.9003	0.4093	0.08908	1.71
$\gamma 16$	0.813	0.4742	-0.1379	1.743
$\gamma 17$	1.076	0.5026	0.07577	2.047
$\gamma 18$	0.8541	0.497	-0.1157	1.836
$\gamma 19$	0.9241	0.4784	-0.01713	1.851
$\gamma 20$	0.8237	0.498	-0.171	1.806
$\gamma 21$	0.9878	0.4799	0.05825	1.945
$\gamma 22$	1.311	0.4235	0.47	2.137
$\gamma 23$	1.105	0.462	0.2004	2.027
$\gamma 24$	0.8266	0.4668	-0.09189	1.75
$\gamma 25$	-0.11	0.3256	-0.7587	0.5301
$\gamma 26$	0.9296	0.4205	0.09995	1.763

$\gamma 27$	0.5301	0.3224	-0.106	1.159
$\gamma 28$	0.496	0.3862	-0.27	1.234
$\gamma 29$	0.7822	0.3686	0.07354	1.511
$\gamma 30$	0.1829	0.1915	-0.1955	0.5617
$\gamma 31$	0.817	0.3549	0.1195	1.527
$\gamma 32$	0.5107	0.3451	-0.1676	1.193
$\gamma 33$	0.3865	0.3091	-0.2382	0.9817
$\gamma 34$	0.4469	0.2277	0.00229	0.8939
$\gamma 35$	0.2592	0.2905	-0.3093	0.8341
$\gamma 36$	0.4108	0.2865	-0.1661	0.9593
$\gamma 37$	0.4485	0.2562	-0.05687	0.9527
$\gamma 38$	0.2712	0.2356	-0.1998	0.7334
$\gamma 39$	0.3043	0.1996	-0.08188	0.7034
$\gamma 40$	0.205	0.1827	-0.152	0.5742
$\gamma 41$	0.4927	0.2444	0.01713	0.9845
$\gamma 42$	0.2894	0.2164	-0.1416	0.7139
$\gamma 43$	-0.0171	0.2364	-0.4891	0.4458
$\gamma 44$	-0.08268	0.2241	-0.5191	0.3615
$\gamma 45$	0.2155	0.1889	-0.1624	0.5829
$\gamma 46$	0.05074	0.2015	-0.345	0.4609
$\gamma 47$	-0.05155	0.1876	-0.4231	0.3197
$\gamma 48$	-0.04876	0.1729	-0.384	0.2919
$\gamma 49$	0.07613	0.186	-0.2926	0.4384
$\gamma 50$	0.005071	0.1837	-0.3599	0.3709
$\gamma 51$	0.06263	0.165	-0.2612	0.3872
$\gamma 52$	-0.1595	0.1582	-0.4726	0.1485
$\gamma 53$	-0.1057	0.1046	-0.3153	0.09565
$\gamma 54$	0.06889	0.143	-0.217	0.3486
Slope May-tempS	mean	sd	2.50%	97.50%
φ	7.71	1.99	2.579	9.937
$1/\sigma^2$ SERE	1.72E-04	3.78E-04	4.33E-07	0.001362

Supplementary Table 7: Analysis of GNES slopes parameter posterior means, SD, and 95%CI.

Parameter	mean	SD	2.5%	97.5%
θ_1 intercept before change point	0.26	0.02	0.20	0.31
θ_2 change in intercept	-0.23	0.02	-0.29	-0.18

$(\theta_1 + \theta_2)$ intercept after change point	0.023	0.007	0.009	0.038
$\theta 3$ slope before change point	-0.75	0.08	-0.91	-0.56
θ_4 change in slope	0.74	0.08	0.56	0.91
$(\theta_3+\theta_4)$ slope after change point	-0.004	0.008	-0.02	0.01
Change point	0.398	0.008	0.385	0.410

Supplementary Table 8: Site locations in degrees for each of the forest stands depicted in Figure S1.

**Site**	**Latitude (◦)**	**Longitude (◦)**
100	44.347	-85.484
20	44.195	-86.092
22	44.373	-85.708
24	44.223	-85.752
3	44.264	-86.178
31	44.272	-85.993
41	44.347	-85.482
50	44.257	-86.084
58	44.310	-85.896
6	44.220	-85.629
7	44.193	-85.677
9	44.321	-85.977

Supplementary References:

1. Zak, D. R. & Pregitzer, K. S. Spatial and Temporal Variability of Nitrogen Cycling in Northern Lower Michigan. *For. Sci.* **36**, 367–380 (1990).
2. Pellitier, P. T., Zak, D. R., Argiroff, W. A. & Upchurch, R. A. Coupled shifts in ectomycorrhizal communities and plant uptake of organic nitrogen along a soil gradient: an isotopic perspective. *Ecosystems* (2021).
3. Taylor, D. L. *et al.* Accurate Estimation of Fungal Diversity and Abundance through Improved Lineage-Specific Primers Optimized for Illumina Amplicon Sequencing. *Appl. Environ. Microbiol.* **82**, 7217–7226 (2016).
4. Callahan, B. J. *et al.* DADA2: High-resolution sample inference from Illumina amplicon data. *Nat. Methods* **13**, 581–583 (2016).
5. Nilsson, R. H. *et al.* The UNITE database for molecular identification of fungi: handling dark taxa and parallel taxonomic classifications. *Nucleic Acids Res.* **47**, D259–D264 (2019).
6. Bokulich, N. A. *et al.* q2-sample-classifier: machine-learning tools for microbiome classification and regression. *J. Open Res. Softw.* **3**, (2018).
7. Garnica, S. *et al.* Determining threshold values for barcoding fungi: lessons from Cortinarius (Basidiomycota), a highly diverse and widespread ectomycorrhizal genus. *FEMS Microbiol. Ecol.* **92**, (2016).
8. Tedersoo, L. & Smith, M. E. Lineages of ectomycorrhizal fungi revisited: Foraging strategies and novel lineages revealed by sequences from belowground. *Fungal Biol. Rev.* **27**, 83–99 (2013).
9. Lodge, D. J. *et al.* Molecular phylogeny, morphology, pigment chemistry and ecology in Hygrophoraceae (Agaricales). *Fungal Divers.* **64**, 1–99 (2014).

10. Agerer, R. Exploration types of ectomycorrhizae. *Mycorrhiza* **11**, 107–114 (2001).

11. Moeller, H. V., Peay, K. G. & Fukami, T. Ectomycorrhizal fungal traits reflect environmental conditions along a coastal California edaphic gradient. *FEMS Microbiol. Ecol.* **87**, 797–806 (2014).

12. Baker, M. E. & King, R. S. A new method for detecting and interpreting biodiversity and ecological community thresholds. *Methods Ecol. Evol.* **1**, 25–37 (2010).

13. Wood, D. E., Lu, J. & Langmead, B. Improved metagenomic analysis with Kraken 2. *Genome Biol.* **20**, 257 (2019).

14. Konar, A. *et al.* High-quality genetic mapping with ddRADseq in the non-model tree Quercus rubra. *BMC Genomics* **18**, 417 (2017).

15. Sork, V. L. *et al.* First Draft Assembly and Annotation of the Genome of a California Endemic Oak. *Genes|Genomes|Genetics* **6**, 3485–3495 (2016).

16. Lombard, V., Golaconda Ramulu, H., Drula, E., Coutinho, P. M. & Henrissat, B. The carbohydrate-active enzymes database (CAZy) in 2013. *Nucleic Acids Res.* **42**, D490–D495 (2014).

17. Fawal, N. *et al.* PeroxiBase: a database for large-scale evolutionary analysis of peroxidases. *Nucleic Acids Res.* **41**, D441–D444 (2013).

18. Treiber, M. L., Taft, D. H., Korf, I., Mills, D. A. & Lemay, D. G. Pre- and post-sequencing recommendations for functional annotation of human fecal metagenomes. *BMC Bioinformatics* **21**, 74 (2020).

19. Li, H. & Durbin, R. Fast and accurate short read alignment with Burrows–Wheeler transform. *Bioinformatics* **25**, 1754–1760 (2009).

20. Kriventseva, E. V. *et al.* OrthoDB v10: sampling the diversity of animal, plant, fungal, protist, bacterial and viral genomes for evolutionary and functional annotations of orthologs. *Nucleic Acids Res.* **47**, D807–D811 (2019).
21. Plummer, M. JAGS: A program for analysis of Bayesian graphical models using Gibbs sampling. *Work. Pap.* 8 (2003).
