# Supplementary material for: Ectomycorrhizal access to organic nitrogen mediates CO2 fertilization response in a dominant temperate tree
Source: Nat Commun. 2021 Sep 13;12:5403. doi: 10.1038/s41467-021-25652-x (PMC8438073; doi:10.1038/s41467-021-25652-x)
Supplement: Supplementary file 3 — Reporting Summary [file 41467_2021_25652_MOESM3_ESM.pdf]

nature research

Corresponding author(s): Peter T. Pellitier & Donald R. Zak

Last updated by author(s): Apr 10, 2021

Reporting Summary

Nature Research wishes to improve the reproducibility of the work that we publish. This form provides structure for consistency and transparency in reporting. For further information on Nature Research policies, see our Editorial Policies and the Editorial Policy Checklist.

Statistics

For all statistical analyses, confirm that the following items are present in the figure legend, table legend, main text, or Methods section.

n/a	Confirmed	
☐	☒	The exact sample size (*n*) for each experimental group/condition, given as a discrete number and unit of measurement
☐	☒	A statement on whether measurements were taken from distinct samples or whether the same sample was measured repeatedly
☐	☒	The statistical test(s) used AND whether they are one- or two-sided<br>*Only common tests should be described solely by name; describe more complex techniques in the Methods section.*
☐	☒	A description of all covariates tested
☐	☒	A description of any assumptions or corrections, such as tests of normality and adjustment for multiple comparisons
☐	☒	A full description of the statistical parameters including central tendency (e.g. means) or other basic estimates (e.g. regression coefficient) AND variation (e.g. standard deviation) or associated estimates of uncertainty (e.g. confidence intervals)
☐	☒	For null hypothesis testing, the test statistic (e.g. *F*, *t*, *r*) with confidence intervals, effect sizes, degrees of freedom and *P* value noted<br>*Give P values as exact values whenever suitable.*
☐	☒	For Bayesian analysis, information on the choice of priors and Markov chain Monte Carlo settings
☒	☐	For hierarchical and complex designs, identification of the appropriate level for tests and full reporting of outcomes
☐	☒	Estimates of effect sizes (e.g. Cohen's *d*, Pearson's *r*), indicating how they were calculated

Our web collection on statistics for biologists contains articles on many of the points above.

Software and code

Policy information about availability of computer code

Data collection	We used Cybis CooRecorder program to measure tree ring widths (Cybis Elektronik Sweden). We then used the program Cybis CDendro for individual cross dating and chronology assembly by site (Cybis Elektronik 2010).
Data analysis	We used the following programs: JAGS 3.4 19 using the rjags package in R for all Bayesian analyses.<br><br>QIIME2 and DADA2 (1.16) were used to analyze fungal sequence amplicon data. Bioinformatic packages used to process metagenomic sequences: BWA-MEM (0.7.17), DIAMOND (0.9.29), Kraken2 (2.0.8), Univec. The R packages, vegan (2.5-6), tidyverse (1.3), TITAN2 (2.4), gdm (1.4.2) were employed in R v. 4.0.2. Scikit naive bayes classiier was employed in QIIME2 (v. 0.21.0).

For manuscripts utilizing custom algorithms or software that are central to the research but not yet described in published literature, software must be made available to editors and reviewers. We strongly encourage code deposition in a community repository (e.g. GitHub). See the Nature Research guidelines for submitting code & software for further information.

Data

Policy information about availability of data

All manuscripts must include a data availability statement. This statement should provide the following information, where applicable:

- Accession codes, unique identifiers, or web links for publicly available datasets
- A list of figures that have associated raw data
- A description of any restrictions on data availability

Raw DNA sequences associated with the ITS2 amplicon sequencing are deposited in NCBI Sequence Read Archive: SRR14164239-SRR14164298. Metagenomic sequences are deposited under accession codes: SRR15377920-SRR15377978. Associated soil metadata are available in Dryad (https://doi.org/ 10.5061/ dryad.4f4qrfjbt). Access to wood cores will be available upon written request. Publicly available datasets used in this study include CAZy and Redoxibase http://

www.cazy.org; http://peroxibase.toulouse.inra.fr/). Univec database: https://ftp.ncbi.nlm.nih.gov/pub/UniVec/. UNITE database: https://unite.ut.ee/. NOAA climatic data https://www.ncdc.noaa.gov/cdo-web]) was used, and in addition, OrthoDB database was used (https://www.orthodb.org/). Finally, the publicly available DEEMY database was accessed at http://www.deemy.de.

Field-specific reporting

Please select the one below that is the best fit for your research. If you are not sure, read the appropriate sections before making your selection.

☐ Life sciences ☐ Behavioural & social sciences ☒ Ecological, evolutionary & environmental sciences

For a reference copy of the document with all sections, see nature.com/documents/nr-reporting-summary-flat.pdf

Ecological, evolutionary & environmental sciences study design

All studies must disclose on these points even when the disclosure is negative.

Study description

The study had three types of related data collection: 1) analyses of tree-cores 2) isolation and analysis of ECM communities using taxonomic meta-barcoding, and shotgun metagenomics 3) Soil analyses as supporting metadata. I provide the detailed methods with sample sizes in the sections below (and where relevant throughout the manuscript). The research took place in a temperate upland forest ecosystem in Manistee National Forest Michigan, USA. and all analyses occurred at the University of Michigan.

Research sample

Increment cores from Quercus rubra L, were obtained from 60 individual trees. Mycorrhizal root-tips comprising a wide-range of taxa were sampled from associated root-systems using (n= 5) 11cm x 11cm square x 10cm deep cores that were composited on the individual tree-basis. Soil samples were obtained from the base of each focal tree-stem and also composited on the individual tree basis. Rationale for sampling was to maintain statistical power at the individual tree basis.

Sampling strategy

In May 2018, at each of the 12 sites we selected five mature Q. rubra individuals that were at least 10-m apart, we measured tree diameter at breast height (1.3 m; DBH) in cm. We then extracted growth cores (to the pith) from the North and South aspect of each tree at DBH using 5.15mm Haglöf increment borers. Soil cores, 5-cm diameter and 10-cm deep, were collected in both May and August 2018. Five soil cores were taken radially around the dripline of each focal Q. rubra individual. Cores were immediately transferred to the laboratory on ice, sieved at 2mm, and homogenized for each tree. Mycorrhizal root-tips comprising a wide-range of taxa were sampled from associated root-systems using (n= 5) 11cm x 11cm square x 10cm deep cores that were composited on the individual tree-basis. Sample sizes were not pre-determined, but sampling size was intended balanced sampling effort on an individual stand basis and also comply with sampling restrictions in the National Forest

Data collection

Data was collected by the following co-authors.
Tree-ring sampling (K.A + I.I). In May 2018, at each of the 12 sites we selected five mature Q. rubra individuals that were at least 10-m apart, we measured tree diameter at breast height (1.3 m; DBH) in cm. We then extracted growth cores (to the pith) from the North and South aspect of each tree at DBH using 5.15mm Haglöf increment borers. The samples were dried overnight at 100° C. We mountemee cores on cradles and progressively sanded them by hand, from 100 to 600 grit. The mounted cores were digitized for measurement by digitizing them on a flatbed scanner at a resolution of 1200 dpi. We measured yearly ring width (growth) of the scanned tree cores using the Cybis CooRecorder program at a precision of 0.001 mm (Cybis Elektronik 2010). We then used the program Cybis CDendro for individual cross dating and chronology assembly by site. Crossdating was achieved when TTest values were greater than 5 for matching target samples using the P2YrsL normalization method67. We created master ring width lists that were summed by stem to reflect the average yearly growth of each individual using cross dated North & South aspects. We estimated historical DBH of focal trees at each year using the yearly ring width from our master chronologies.

Field Sampling (P.T.P, D.R.Z, W.A, K.A): Listed individuals participated in the collection of materials. Each member collected both tree-core, fungal and soil samples.

Soil Analyses (P.T.P): Soil cores, 5-cm diameter and 10-cm deep, were collected in both May and August 2018. Five cores were taken radially around the dripline of each focal Q. rubra individual. Cores were immediately transferred to the laboratory on ice, sieved at 2mm, and homogenized for each tree. Soil net N mineralization rates were quantified as an estimate of soil inorganic N availability for soil samples collected in both May and August 2018. Soil inorganic N was extracted from fresh sieved soil using 2M KCl, followed by a 14-day aerobic incubation in order to measure rates of soil inorganic N mineralization. NO3- and NH4+ in soil extracts were analyzed colorometrically. Total free primary amines (TFPA) in soil (primarily amino acids and amino sugars) was measured using fresh sieved soil extracted with 2M KCl, extracts used for this purpose were identical to those used to measure extractable NO3- and NH4+ prior to aerobic incubation. TFPA is expressed as umol leucine equivalents, because leucine was used as the analytical standard (Fig. S9); estimates of TFPA availability may be considered relative indices of labile organic N availability in soil solution which is distinct from N-SOM. Total C and N contents of dried sieved bulk soil (% of dry mass) were determined using combustion analysis on a LECO TruMac CN analyzer for soil collected in May 2018. Soil pH was determined for 2:1 deionized water-soil slurries with an Accumet 15 pH meter for soil collected in August 2018.

DNA extractions, PCR, bioinformatics (P.T.P):
In August 2018, ECM root-tips were collected radially around the dripline of each focal Q. rubra individual; five cores were taken around each tree, each core was 10-cm deep and 11x11 cm in area. Cores were obtained immediately adjacent to the soil cores described above. ECM cores were pooled for each individual focal tree and within 12 days of sampling, definitive ECM root-tips with high turgor were manually excised using a dissecting microscope after visually eliminating non-Quercus roots. Sampling was standardized by visually assessing the tips of ~ 90% (wet weight) of all Quercus roots in each of the root cores. In total, 14,944 individual ECM root-tips were excised. DNA was extracted from lyophilized root-tips using the Qiagen DNeasy Plant Mini Kit (Hilden, Germany) and DNA pools were split for amplicon and metagenomic sequencing. The ITS2 fragment of rRNA was amplified using PCR,

following Taylor et al. 2016 and sequenced using Illumina Mi-Seq (2 x250; San Diego, CA). Metagenomic sequence libraries were prepared as detailed, by the University of Michigan Advanced Genomics Core. Processing of metagenomic sequence data data was conducted by P.T.P. Specific details on DNA extractions, PCR, library preparation are reported in full in the supplement.

Bayesian modeling of tree-core data: I.I.

Timing and spatial scale

Soil and tree-ring sampling in May and August 2018 was achieved over the course of five days during daylight hours. The spatial scale of data collection is at the level of the individual tree stem. Soil samples were obtained at the drip-line of each Quercus rubra individual, and range from 2-4m from the trunk. Sites were visited in a randomized manner, so as not to bias sampling date with properties of the soil gradient. Samples were stored on ice while in the field prior to transferring them to the lab. Sampling in August was the peak of the growing season, and represented plausible maximum photosynthate allocation belowground. Rationale for sampling in Manistee National Forest is primarily based on known disturbance history and several decades of local knowledge garnered working in these stands.

Data exclusions

No samples were removed a priori. One sample was removed due to few quality amplicon sequences. This sample was then omitted from all subsequent analyses. One sample did not yield a usable library for metagenomic sequencing and was thus removed from all subsequent analyses. Six individual trees did not yield usable tree-ring data, and these samples were removed. These omissions are noted in the main text and supplement

Reproducibility

Field Sampling: We collected a large dataset of soil fungal communities. Parameters employed are reported alongside each software package version. Soil sampling efforts cannot be reproduced due to destructing sampling. Results were primarily observational

Randomization

Geographic covariates are incorporated into appropriate models for dendrochronological and multivariate (fungal) analyses. All other covariates are reported

Blinding

None used in this observational study

Did the study involve field work? ☒ Yes ☐ No

Field work, collection and transport

Field conditions

Soil samples for DNA extraction were obtained during the growing season in August 2018 when photosynthate allocation belowground is likely greatest. Tree-cores were obtained in May of 2018, during the commencement of the growing season. Our study took place across a regional network of 12 forest sites comprising a known natural nitrogen availability soil gradient. Daytime temperatures in May 2018 ranged from 20-22C, with no precipitation the preceding week or during sampling. Sampling in August 2018: temperatures ranged from 22-28C, with no precipitation the preceding week or during sampling. Samples were collected from 8am-4pm.

Location

Manistee National Forest, Wexford County Michigan, USA. Elevation ranged from 70-130m. Coordinates for each site are reported as a supplementary table (S7).

Access & import/export

Sampling agreements were obtained from the U.S. Forest Service by DR. Zak.

Disturbance

Disturbances incurred upon sampling include soil collection, and tree coring. Tree-cores were of small scale, and wound recovery spray was added after removal of core. Soil samples were of minimal size and balanced disturbance with representative sampling of fungal communities. Trees are tagged in the field so as to identify subsequent researchers of disturbance incurred for the current study.

Reporting for specific materials, systems and methods

We require information from authors about some types of materials, experimental systems and methods used in many studies. Here, indicate whether each material, system or method listed is relevant to your study. If you are not sure if a list item applies to your research, read the appropriate section before selecting a response.

Materials & experimental systems

n/a	Involved in the study
☒	☐ Antibodies
☒	☐ Eukaryotic cell lines
☒	☐ Palaeontology and archaeology
☒	☐ Animals and other organisms
☒	☐ Human research participants
☒	☐ Clinical data
☒	☐ Dual use research of concern

Methods

n/a	Involved in the study
☒	☐ ChIP-seq
☒	☐ Flow cytometry
☒	☐ MRI-based neuroimaging
